# The comparative effects of oral Chinese patent medicines in non-proliferative diabetic retinopathy: A Bayesian network meta-analysis of randomized controlled trials

**DOI:** 10.3389/fendo.2023.1144290

**Published:** 2023-04-03

**Authors:** Ziqiang Liu, Yunru Chen, Chuanhong Jie, Jianwei Wang, Yu Deng, Xiaoyu Hou, Yuanyuan Li, Wenjing Cai

**Affiliations:** ^1^ Ophthalmology Department, Eye Hospital China Academy of Chinese Medical Sciences, Beijing, China; ^2^ Centre for Evidence-based Chinese Medicine, School of Traditional Chinese Medicine, Beijing University of Chinese Medicine, Beijing, China

**Keywords:** oral Chinese patent medicines, non-proliferative diabetic retinopathy, calcium dobesilate, network meta-analysis, SUCRA

## Abstract

**Background:**

Non-proliferative diabetic retinopathy (NPDR), a common diabetic complication with high morbidity, is featured by impaired visual function and fundus lesions. It has been reported that oral Chinese patent medicines (OCPMs) may improve visual acuity and fund signs. However, the best possible OCPMs for NPDR remain questionable and merit further investigation.

**Methods:**

From inception to October 20, 2022, seven databases were searched for eligible randomized controlled trials (RCTs). The outcomes were clinical effective rate, visual acuity, visual field gray value, microaneurysm volume, hemorrhage area, macular thickness, and adverse events rate. The revised Cochrane risk-of-bias tool (ROB 2) was used to assess the quality of the included studies. Network meta-analysis was performed using R 4.1.3 and STATA 15.0 software.

**Results:**

We included 42 RCTs with 4,858 patients (5,978 eyes). The Compound Danshen Dripping Pill (CDDP) combined with calcium dobesilate (CD) had the most improvement in clinical efficacy rate (SUCRA, 88.58%). The Compound Xueshuantong Capsule (CXC) combined with CD may be the best intervention (SUCRA, 98.51%) for the improvement of visual acuity. CDDP alone may be the most effective treatment option (SUCRA, 91.83%) for improving visual field gray value. The Hexuemingmu Tablet (HXMMT) and Shuangdan Mingmu Capsule (SDMMC) combined with CD may be the most effective treatment for reducing microaneurysm volume and hemorrhage area (SUCRA, 94.48%, and 86.24%), respectively. Referring to reducing macular thickness, CXC combined with CD ranked first (SUCRA, 86.23%). Moreover, all OCPMs did not cause serious adverse reactions.

**Conclusion:**

OCPMs are effective and safe for NPDR. CDDP alone, and combined with CD, may be the most effective in improving visual field gray value and clinical efficacy rate, respectively; CXC combined with CD may be the best in enhancing BCVA and reducing macular thickness; HXMMT and SDMMC combined with CD, maybe the most effective regarding microaneurysm volume and hemorrhage area, respectively. However, the reporting of methodology in the primary study is poor, potential biases may exist when synthesizing evidence and interpreting the results. The current findings need to be confirmed by more large-sample, double-blind, multi-center RCTs of rigorous design and robust methods in the future.

**Systematic review registration:**

https://www.crd.york.ac.uk/prospero/, identifier CRD42022367867.

## 1 Introduction

The prevalence of diabetes in individuals aged 20 to 79 years increased to 537 million worldwide in 2021 and is expected to rise to 783 million in 2045 (1). Diabetic retinopathy (DR) is one of the most common microvascular and neurological complications of diabetes, with a prevalence of 24.7-37.5% in the diabetic population, and is the leading cause of blindness in people of working age (2). According to the international clinical DR severity grading criteria, DR can be divided into non-proliferative diabetic retinopathy (NPDR) and proliferative diabetic retinopathy (PDR). NPDR is characterized by fundus microaneurysms, retinal hemorrhage, cotton wool spots, hard exudate, and intraretinal microvascular abnormality (IRMA) formation. Moreover, retinal neovascularization formation implies a transition to the PDR stage, which can further cause vitreous hemorrhage, retinal detachment, and neovascular glaucoma, leading to severe vision loss and even blindness (3).

Therefore, controlling the progression of DR and maximizing the restoration of visual acuity in patients has become a focus of clinical research. Currently, the main treatment strategies for NPDR are controlling risk factors, such as blood glucose and blood pressure, and improving microcirculation. Retinal laser photocoagulation, intravitreous drug injection, and vitrectomy are mainly applied to patients with PDR, and all have some limitations (4). Calcium dobesilate (CD) is a well-established vasoactive and vasoprotective drug that can reduce retinal capillary permeability and stabilize blood-retinal barrier function, as well as antagonize platelet aggregation and improve local blood circulation, and is widely used clinically in patients with NPDR (5, 6). A meta-analysis involving 221 studies showed that CD might improve fundus bleeding and exudation in patients with DR (5). However, not all NPDR patients benefit (7).

In addition to the above methods, Chinese clinicians have achieved better clinical efficacy by combining oral Chinese patent medicines (OCPMs). OCPMs are traditional Chinese medicine products processed as per the prescription and preparation technology based on Chinese herbal medicine as raw material (8). Several recent basic studies have shown that the active ingredients of OCPMs can reduce retinal ischemia and hypoxia, and improve retinal structure and function through various pathways, such as reducing pericyte loss, attenuating oxidative stress and inflammatory responses (9, 10). Moreover, a growing body of clinical evidence suggested that OCPMs alone or combined with CD for treating NPDR patients could effectively improve patients’ visual function and fundus signs (11). However, there is still debate about which OCPM is most effective in treating NPDR. Therefore, our study aimed to systematically assess the effects of different OCPMs on outcome indicators of NPDR using network meta-analysis (NMA).

## 2 Methods

The study followed the Preferred Reporting Items for Systematic Reviews and Meta-Analyses (PRISMA) Extension Statement for systematic reviews and meta-analyses (12). The PRISMA checklist is detailed in **Supplementary File S1**.

### 2.1 Search strategy

Seven academic databases were searched for published research from inception to October 20, 2022, including PubMed, Embase, Cochrane Library, China National Knowledge Infrastructure, Wanfang Database, Weipu Journal Database, and Chinese Biomedical Literature Database. Only Chinese and English articles were retrieved. The detailed search strategies are provided in **Supplementary File S2**.

### 2.2 Inclusion and exclusion criteria

1) Patients diagnosed with NPDR according to international or Chinese diagnostic criteria (relying on fundus fluorescein angiography and fundus signs) (3, 13).
2) The interventions of the experimental group were OCPMs with or without CD. Besides, the OCPMs must belong to the seven kinds of OCPMs recommended by the National Healthcare Security Administration (NHSA) (http://www.nhsa.gov.cn/) and National Medical Products Administration (NMPA) (https://www.nmpa.gov.cn/) of the People’s Republic of China.
3) The interventions of the control group were treated with CD alone.
4) Outcomes included clinical effective rate (percentage of patients whose visual acuity and fund signs improved after treatment), visual acuity (standard logarithm visual acuity chart), visual field gray value, microaneurysm volume, hemorrhage area, macular thickness, and adverse drug reactions (ADRs).

5) The types of studies were randomized controlled trials (RCTs).

### 2.3 Data collection and quality assessment

Two authors independently extracted the relevant information. The details included basic trial information, population, detailed inventions, outcomes, and study type. According to Version 2 of the Cochrane risk-of-bias tool (RoB 2) for randomized trials, Two authors independently assessed the risk of bias of the RCTs from five submissions, including randomization process, deviations from intended interventions, missing outcome data, measurement of the outcome, and selection of the reported results (14). All trials were regarded as “low risk”, “some concerns”, or “high risk”. Disagreements were resolved through consensus or third-party adjudication.

### 2.4 Data analysis

We used STATA 15.0 software to conduct a traditional pairwise meta-analysis and R 4.1.3 software with the “BUGSnet” and “rjags” packages for a Bayesian NMA (15). A random-effects model was analyzed. We ran the Markov chain Monte Carlo (MCMC) simulation with four Markov chains for 200,000 iterations (burn-in iterations=5000, thinning factor=1) (16). The Gelman-Rubin convergence diagnostic was tested. The potential scale reduction factor (PSRF) value close to 1 indicates convergence. We estimated the odds ratio (OR) for dichotomous outcomes and mean difference (MD) for continuous outcomes, with a corresponding 95% credible interval (CrI). The network plot was presented to visualize multiple comparisons. The evaluation of inconsistency was not applicable because there were no “closed loops” in the network plot. The probability values of the surface under the cumulative ranking curve (SUCRA) were estimated for treatment rankings. The SUCRA values ranged from 0-100%. A higher value indicates a higher likelihood that therapy is the best among the interventions being compared (17). Heterogeneity was assessed using the I^2^ test. If there was substantial heterogeneity (I^2^ > 50%), subgroup analysis and sensitivity analysis were considered. Publication bias was examined by the comparison-adjusted funnel plot.

## 3 Results

### 3.1 Search results

**Figure 1:**
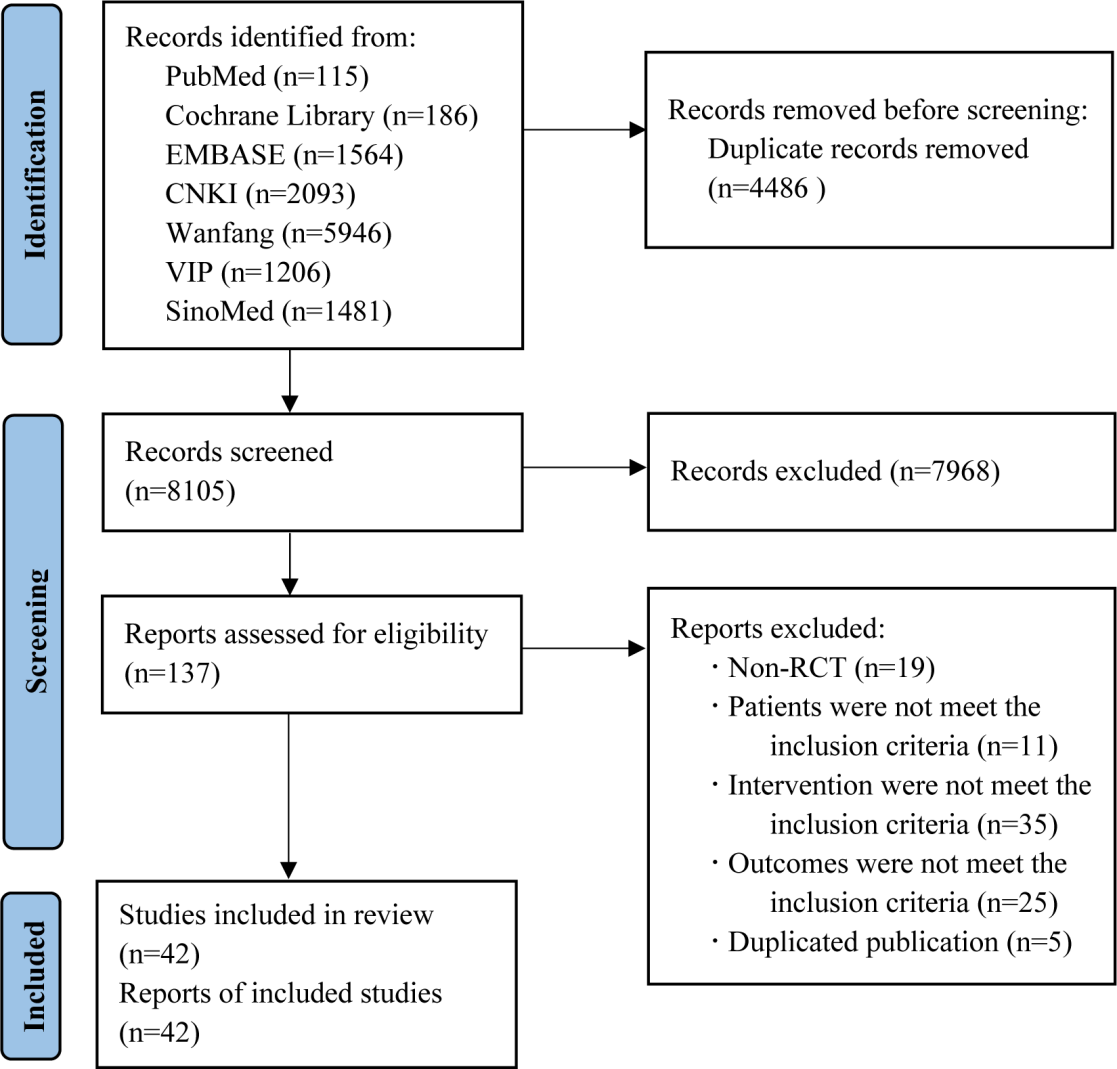
Flowchart of the search for eligible studies.

We retrieved 12,591 records in total. After removing duplicates, 8,105 records remained for screening. Of which, 7,968 records were excluded by reading the title and abstract, and 95 by reading the full text. Finally, 42 two-arm RCTs with 4,858 patients (5,978 eyes) were included in our study. The Flowchart of the search is shown in **Figure 1**.

### 3.2 Characteristics of included studies

In total, 4,858 patients (5,978 eyes) and 7 kinds of OCPMs were involved in the 42 RCTs. Concerning treatment, 2,334 patients (2,790 eyes) used CD alone, 846 patients (1,351 eyes) treated only OCPMs, and 1,678 patients (1,837 eyes) received with OCPMs combined with CD. Regarding outcomes, 36 studies (85.71%), 8 studies (19.05%), 19 studies (45.24%), 14 studies (33.33%), 18 studies (42.86%), 18 studies (42.86%), and 22 studies (52.38%) assessed the clinical efficacy rate, visual acuity, visual field gray value, microaneurysm volume, hemorrhage area, macular thickness, and ADRs, respectively. The detailed characteristics of the studies are demonstrated in **Table 1**.

**Table 1:** Characteristics of the included studies.

Study ID	Sample Size (E/C)	Sex (M/F)	Age (Year, E/C)	Intervention of experimental group	Intervention of control group	Course (Months)	Outcomes
Du JH 2018 (18)	48/48	(29/19)/(30/18)	(64.2 ± 4.3)/(62.7 ± 4.0)	CXC	CD	1	①
Huang W 2021 (19)	30/30	(17/13)/(18/12)	(52.85 ± 6.38)/(51.86 ± 6.16)	CXC+CD	CD	5	① ③ ④ ⑤ ⑥ ⑦
An LN 2020 (20)	35/35	(19/16)/(20/15)	(51.17 ± 17.83)/(52.12 ± 15.76)	CXC+CD	CD	3	① ④ ⑥ ⑦
Wang J 2020 (21)	44/42	(26/19)/(23/19)	(69.52 ± 7.11)/(68.35 ± 6.82)	CXC+CD	CD	5	① ③ ④ ⑤ ⑥ ⑦
Yan H 2020 (22)	46/46	(27/19)/(25/21)	(48.5 ± 4.9)/(47.4 ± 4.6)	CXC+CD	CD	3	① ⑦
Chai F 2018 (23)	54/53	(32/22)/(30/23)	(61.11 ± 6.01)/(61.19 ± 6.03)	CXC+CD	CD	3	① ③ ⑤ ⑥
Wang Q 2018 (24)	50/50	(26/24)/(27/23)	(55.02 ± 2.58)/(54.80 ± 2.61)	CXC+CD	CD	6	① ③ ④ ⑤ ⑥
Ma JP 2018 (25)	27/27	(16/11)/(15/12)	(53.02 ± 4.13)/(53.08 ± 4.25)	CXC+CD	CD	5	① ③ ④ ⑤ ⑥ ⑦
Yu W 2017 (26)	34/34	(19/15)/(17/17)	(57.4 ± 8.3)/(58.1 ± 7.9)	CXC+CD	CD	3	① ③ ⑤ ⑥
Rao XJ 2017 (27)	110/125	(61/49)/(68/57)	(49.5 ± 5.9)/(50.2 ± 6.4)	CXC+CD	CD	3	①
Men LB 2020 (28)	40/40	(21/19)/(22/18)	(66.97 ± 2.86)/(67.46 ± 2.52)	CXC+CD	CD	2	① ② ③ ⑤ ⑥
Li Y 2019 (29)	49/49	(28/21)/(27/22)	(66.82 ± 4.03)/(66.41 ± 4.11)	CXC+CD	CD	3	① ③
Pei R 2015 (30)	32/32	(17/15)/(16/16)	(56.4 ± 2.1)/(55.3 ± 1.2)	CXC+CD	CD	5	① ③ ④ ⑤ ⑥ ⑦
Zhou YD 2022 (31)	63/63	(34/29)/(35/28)	(51.14 ± 8.1)/(52.04 ± 2.2)	CXC+CD	CD	3	① ③ ④ ⑤ ⑥ ⑦
Luo D 2015 (32)	28/29	(18/10)/(19/10)	(59.54 ± 7.46)/(57.86 ± 10.03)	CDDP	CD	3	③
Jin M 2009 (33)	30/28	NR	(62.78 ± 7.69)/(61.11 ± 7.27)	CDDP	CD	3	② ③ ⑤
Chen Y 2006 (34)	31/32	(17/14)/(15/17)	(54.60 ± 10.40)/(58.12 ± 9.31)	CDDP	CD	3	①
Xu HT 2019 (35)	43/43	(24/19)/(25/18)	(53.11 ± 4.41)/(53.06 ± 4.39)	CDDP+CD	CD	4	① ③ ④ ⑤ ⑥ ⑦
Li Y 2017 (36)	89/89	(31/58)/(28/61)	(56.5 ± 7.2)/(55.8 ± 6.8)	CDDP+CD	CD	2	①
Wang HM 2016 (37)	45/45	(23/22)/(24/21)	(57.15 ± 6.68)/(57.06 ± 6.72)	CDDP+CD	CD	2	①
Bai YX 2017 (38)	38/38	(20/18)/(21/17)	(40-72)/(39-71)	CDDP+CD	CD	4	① ② ③ ④ ⑤ ⑦
Ruan YX 2017 (39)	35/35	(18/17)/(20/15)	(52.5 ± 1.1)/(52.8 ± 1.7)	CDDP+CD	CD	4	① ③ ④ ⑤ ⑥ ⑦
Huang YX 2021 (40)	45/45	(28/17)/(29/16)	(67.5 ± 5.3)/(67.3 ± 5.1)	CDDP+CD	CD	6	② ③ ④ ⑦
Qin YH 2010 (41)	414/221	NR	NR	SDMUC	CD	4	① ⑦
Ji XD 2022 (42)	52/52	(29/23)/(28/24)	(56.63 ± 4.02)/(56.53 ± 4.09)	SDMUC+CD	CD	3	① ⑤ ⑥ ⑦
Liu JP 2019 (43)	60/60	(33/27)/(32/28)	(57.54 ± 8.11)/(57.10 ± 9.26)	SDMUC+CD	CD	4	① ⑤ ⑥ ⑦
Jin L 2019 (44)	72/71	(44/28)/(43/28)	(63.07 ± 8.08)/(62.39 ± 8.34)	SDMUC+CD	CD	4	⑦
Pang YH 2015 (45)	40/40	(18/22)/(16/24)	(49.4 ± 5.7)/(49.6 ± 5.3)	SDMUC+CD	CD	4	①
Zhang DX 2015 (46)	60/59	(24/36)/(24/35)	(60.03 ± 6.11)/(60.79 ± 642)	QG	CD	3	①
Fang J 2022 (47)	51/51	(27/24)/(26/25)	(45.5 ± 1.3)/(50.0 ± 1.4)	QG	CD	6	① ⑦
Duan JG 2006 (48)	107/105	NR	NR	QG	CD	3	⑦
Fan YP 2018 (49)	47/47	(26/21)/(27/20)	(48.6 ± 5.1)/(47.5 ± 4.9)	QG	CD	6	① ⑥ ⑦
Feng JL 2016 (50)	42/41	(29/13)/(27/14)	(55.26 ± 6.29)/(55.89 ± 6.13)	QG+CD	CD	3	① ②
Wang ZQ 2019 (51)	52/48	(31/21)/(29/19)	(66.7 ± 6.2)/(66.8 ± 6.3)	QG+CD	CD	6	①
Wang ZZ 2017 (52)	47/47	(29/18)/(26/21)	(54.5 ± 4.8)/(54.3 ± 4.9)	QG+CD	CD	3	⑦
Sui HL 2014 (53)	43/43	(22/21)/(23/20)	(50.22 ± 14.82)/(50.53 ± 11.28)	QG+CD	CD	6	① ②
Yan JH 2020 (54)	41/41	(24/17)/(25/16)	(56.65 ± 4.02)/(56.96 ± 4.59)	QG+CD	CD	2	① ② ⑦
Ye XL 2019 (55)	88/88	(46/42)/(50/38)	(60.5 ± 13.4)/(60.9 ± 12.7)	HXMMT+CD	CD	3	①
Gao L 2020 (56)	128/128	(75/53)/(72/56)	(58.14 ± 7.63)/(57.65 ± 7.82)	HXMMT+CD	CD	3	① ③ ④ ⑤ ⑥ ⑦
Zhu HM 2013 (57)	30/30	(13/17)/(14/16)	(61.5 ± 13.1)/(61.6 ± 12.7)	DHHYK	CD	3	① ②
Li JB 2019 (58)	54/54	(29/25)/(28/26)	(55.4 ± 3.1)/(56.1 ± 3.7)	MMDHP+CD	CD	5	① ③ ④ ⑤ ⑥ ⑦
A YN 2019 (59)	50/50	(20/30)/(22/28)	(52.61 ± 5.39)/(53.02 ± 5.41)	MMDHP+CD	CD	1	③ ④ ⑤ ⑥

E/C, experimental group/control group; M/F, male/female; OCPMs, oral Chinese patent medicines; NR, Not Reported; CD, calcium dobesilate; CXC, Compound Xueshuantong Capsule; CDDP, Compound Danshen Dripping Pill; SDMUC, Shuangdan Mingmu Capsule; QG, Qiming Granule; HXMMT, Hexuemingmu Tablet; DHHYK, Danhong Huayu Koufuye; MMDHP, Mingmu Dihuang Pill;   ①Clinical effective rate;   ②Visual acuity;   ③visual field gray value;   ④microaneurysm volume;   ⑤hemorrhage area;   ⑥macular thickness;   ⑦adverse events rate

**Figure 2:**
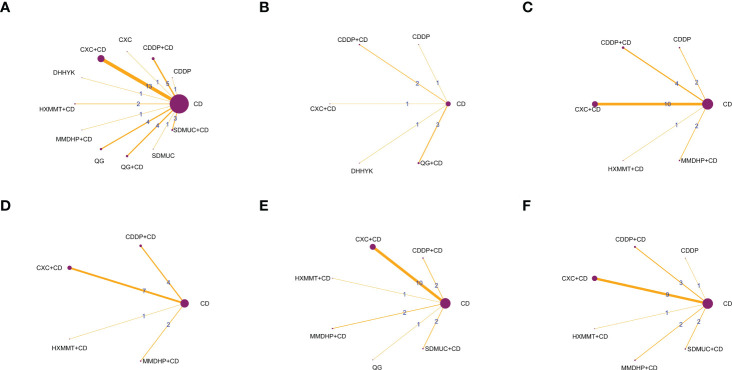
The network graphs comparing OCPMs for NPDR. **(A)** Clinical effective rate; **(B)** Visual acuity; **(C)** Visual field gray value; **(D)** Microaneurysm volume; **(E)** Hemorrhage area; **(F)** Macular thickness. CD, calcium dobesilate; CXC, Compound Xueshuantong Capsule; CDDP, Compound Danshen Dripping Pill; SDMUC, Shuangdan Mingmu Capsule; QG, Qiming Granule; HXMMT, Hexuemingmu Tablet; DHHYK, Danhong Huayu Koufuye; MMDHP, Mingmu Dihuang Pill.

All 42 included RCTs were two-arm studies. The interventions of the experimental group were either OCMPs alone or OCMPs combined with CD, and the control group was CD alone. There were six different types of OCMPs among the combined therapies, including the Compound Xueshuantong Capsule (CXC) combined with CD [13 RCTs (19–31)], the Compound Danshen Dripping Pill (CDDP) combined with CD [6 RCTs (35–40)], the Shuangdan Mingmu Capsule (SDMUC) combined with CD [4 RCTs (42–45)], the Qiming Granule (QG) combined with CD [5 RCTs (50–54)], the Hexuemingmu Tablet (HXMMT) combined with CD [2 RCTs (55, 56)], and the Mingmu Dihuang Pill (MMDHP) combined with CD [2 RCTs (58, 59)]. There were five different kinds of OCMPs among using OCMPs alone, including CXC [only 1 RCT (18)], CDDP [3 RCTs (32–34)], SDMUC [1 RCT (41)], QG [4 RCTs (46–49)] and Danhong Huayu Koufuye (DHHYK) [only 1 RCT (57)]. Detailed information OCMPs is described in **Supplementary File S3**. Furthermore, a network graph depicted the relationship between various interventions and each outcome, which is shown in **Figure 2**.

### 3.3 Risk of bias assessment

**Figure 3:**
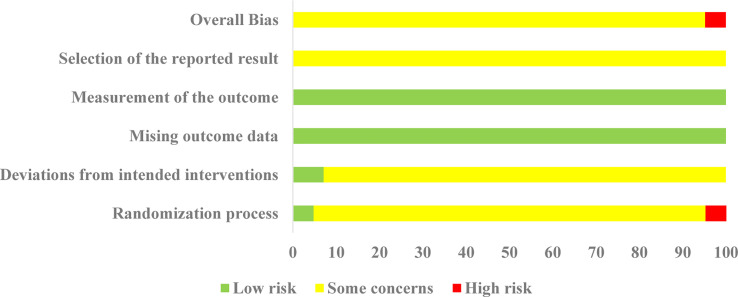
Summary of risk of bias assessment based on revised Cochrane RoB 2 tool.

All 42 selected RCTs reported specific randomization methods, including 18 RCTs (19, 21, 22, 24–26, 28, 29, 31, 35, 38, 40, 43, 48, 49, 55–57) using a simple random number **Table 1** RCT (52) using the stratified randomization, and 1 RCT (41) using random parallel control method. Two RCTs were regarded as “low risk” in the “randomization process” due to reporting allocation concealment. Two RCTs (27, 39) were classified as high-risk because they were randomized by order of visit and admission number. In terms of deviations from intended interventions, three RCTs (32, 41, 48) adopted double-blind methods, and were thus considered as “low risk”, and all the other RCTs were rated as “some concerns”. All studies were evaluated as “low risk” in the missing outcome data due to there were complete data in all studies; In terms of measurement of the outcome, all studies were rated as “low risk” because the two groups were consistent and objective; In addition, there were no details of registration reported or any previously published study protocols, so all RCTs were rated as “some concerns”. Thus, apart from two RCTs (27, 39), all RCTs were rated as “some concerns”. The details of the risk of bias assessment are depicted in **Figure 3**.

### 3.4 Pairwise meta-analysis

We performed the pairwise meta-analysis in the seven outcomes with different OCMPs with or without CD versus CD in NPDR patients. The results (forest plots and heterogeneity analysis) are shown in **Supplementary File S4**. Overall, the heterogeneity of direct comparisons was moderate (I^2^ < 50% for most comparisons), except QG compared to CD for the clinical efficacy rate (I^2^ = 83.0%), CXC+CD compared to CD for the ray value of the visual field (I^2^ = 96.2%), CXC+CD compared to CD for macular thickness (I^2^ = 96.2%). Therefore, we used the fixed-effects model. Since the patients with DR included in this study were all staged as NPDR, which was consistent and had no obvious clinical heterogeneity, different courses of treatment may be substantial sources of clinical heterogeneity. Through different courses of OCMPs, we conducted subgroup analysis to find the source of heterogeneity. Meanwhile, after changing the effect model and eliminating the literature effect size one by one, the original results remained unchanged, indicating that the sensitivity analysis results were negative and the results were relatively reliable. The details are shown in **Supplementary Files S5–S7**.

### 3.5 Network meta-analysis

#### 3.5.1 Clinical efficacy clinical rate

**Figure 4:**
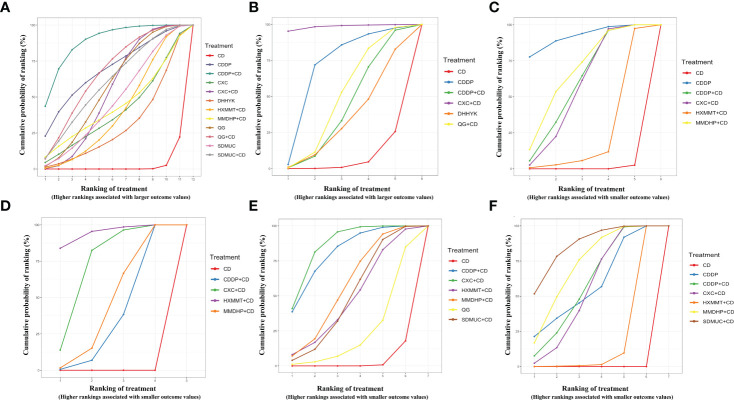
Surface under the cumulative ranking curve (SUCRA) probabilities of different interventions for six outcomes. **(A)** Clinical effective rate; **(B)** Visual acuity; **(C)** Visual field gray value; **(D)** Microaneurysm volume; **(E)** Hemorrhage area; **(F)** Macular thickness. CD, calcium dobesilate; CXC, Compound Xueshuantong Capsule; CDDP, Compound Danshen Dripping Pill; SDMUC, Shuangdan Mingmu Capsule; QG, Qiming Granule; HXMMT, Hexuemingmu Tablet; DHHYK, Danhong Huayu Koufuye; MMDHP, Mingmu Dihuang Pill.

Thirty-six studies reported the clinical efficacy rate. We found that all the included OCPMs, apart from CXC, DHHYK, and MMDHP+CD had higher clinical efficacy than CD alone (**Table 2A**). Based on the ranking probability of SUCRA, CDDP+CD had the highest efficacy rate (88.58%), followed by CDDP (69.27%) and QG+CD (66.98%), whereas CD alone obtained the worst effect (2.27%). The detail is shown in **Figure 4A** and **Table 3**.

**Table 2:** League table of NMA estimations.

(A) Network meta-analysis comparisons for clinical effective rate																						
CD																						
**0.16 (0.03,0.69)**	**CDDP**																					
**0.11 (0.04,0.26)**	**0.66 (0.11,4.09)**				**CDDP+CD**																	
0.33 (0.07,1.43)	2.05 (0.24,17.82)				3.11 (0.53,18.86)	**CXC**																
**0.24 (0.15,0.36)**	1.45 (0.31,7.62)				2.20 (0.82,6.73)	0.71 (0.15,3.51)			**CXC+CD**													
0.41 (0.11,1.47)	2.53 (0.36,19.87)				3.85 (0.81,20.21)	1.24 (0.17,9.20)			1.73 (0.45,6.89)			**DHHYK**										
**0.31 (0.13,0.70)**	1.88 (0.35,11.58)				2.85 (0.86,10.97)	0.92 (0.17,5.29)			1.29 (0.51,3.40)			0.74 (0.16,3.50)			**HXMMT+CD**							
0.31 (0.05,1.63)	1.92 (0.18,19.29)				2.92 (0.37,20.89)	0.93 (0.09,9.07)			1.32 (0.20,7.41)			0.75 (0.08,6.25)			1.01 (0.13,6.54)			**MMDHP+CD**				
**0.23 (0.10,0.42)**	1.38 (0.26,7.58)				2.09 (0.66,6.94)	0.68 (0.13,3.50)			0.95 (0.41,2.04)			0.55 (0.12,2.24)			0.74 (0.23,2.03)			0.72 (0.11,5.00)	**QG**			
**0.19 (0.08,0.42)**	1.15 (0.21,6.93)				1.74 (0.50,6.53)	0.56 (0.10,3.20)			0.79 (0.30,2.00)			0.45 (0.10,2.10)			0.61 (0.18,1.94)			0.60 (0.09,4.53)	0.83 (0.29,2.54)	**QG+CD**		
**0.27 (0.09,0.77)**	1.66 (0.27,11.24)				2.50 (0.66,11.20)	0.81 (0.13,5.18)			1.13 (0.38,3.66)			0.65 (0.12,3.49)			0.88 (0.23,3.39)			0.86 (0.12,7.22)	1.18 (0.37,4.59)	1.44 (0.39,5.65)	**SDMUC**	
**0.21 (0.07,0.56)**	1.28 (0.21,8.48)				1.95 (0.47,8.20)	0.62 (0.10,3.91)			0.88 (0.27,2.65)			0.50 (0.09,2.59)			0.68 (0.17,2.50)			0.67 (0.09,5.43)	0.93 (0.27,3.25)	1.12 (0.28,4.22)	0.77 (0.17,3.25)	**SDMUC+CD**
(B) Network meta-analysis comparisons for visual acuity																						
**CD**																						
-0.19 (-0.41,0.03)	**CDDP**																					
-0.09 (-0.25,0.05)	0.10 (-0.17,0.36)				**CDDP+CD**																	
**-0.46 (-0.68,-0.24)**	-0.27 (-0.58,0.04)				**-0.37 (-0.62,-0.10)**	**CXC+CD**																
-0.07 (-0.29,0.15)	0.12 (-0.19,0.43)				0.02 (-0.23,0.29)	**0.39 (0.08,0.70)**			**DHHYK**													
-0.11 (-0.24,0.02)	0.08 (-0.17,0.33)				-0.02 (-0.21,0.18)	**0.35 (0.10,0.60)**			-0.04 (-0.29,0.21)			**QG+CD**										
(C) Network meta-analysis comparisons for visual field gray value																						
**CD**																						
**1.29 (0.73,1.81)**	**CDDP**																					
**0.93 (0.67,1.19)**	-0.36 (-0.93,0.27)			**CDDP+CD**																		
**0.92 (0.75,1.08)**	-0.37 (-0.91,0.21)			-0.02 (-0.32,0.29)		**CXC+CD**																
0.51 (-0.00,1.02)	**-0.78 (-1.50,-0.01)**			-0.42 (-1.00,0.15)		-0.41 (-0.95,0.13)			**HXMMT+CD**													
**0.99 (0.63,1.35)**	-0.30 (-0.92,0.37)			0.06 (-0.39,0.50)		0.07 (-0.33,0.47)			0.48 (-0.15,1.11)			**MMDHP+CD**										
(D) Network meta-analysis comparisons for microaneurysm volume																						
**CD**																						
**3.05 (2.56,3.51)**	**CDDP+CD**																					
**3.52 (3.05,3.99)**	0.47 (-0.19,1.15)			**CXC+CD**																		
**4.04 (3.14,4.95)**	0.99 (-0.01,2.03)			0.53 (-0.49,1.54)			**HXMMT+CD**															
**3.17 (2.66,3.65)**	0.12 (-0.56,0.81)			-0.35 (-1.05,0.33)			-0.87 (-1.92,0.14)			**MMDHP+CD**												
(E) Network meta-analysis comparisons for hemorrhage area																						
**CD**																						
**0.80 (0.50,1.10)**		**CDDP**																				
**0.81 (0.69,0.96)**		0.01 (-0.31,0.35)	**CDDP+CD**																			
**0.81 (0.73,0.90)**		0.01 (-0.30,0.32)	-0.01 (-0.17,0.15)					**CXC+CD**														
**0.56 (0.38,0.74)**		-0.24 (-0.59,0.11)	**-0.25 (-0.50,-0.04)**					**-0.25 (-0.46,-0.05)**			**HXMMT+CD**											
**0.85 (0.73,0.99)**		0.05 (-0.27,0.38)	0.04 (-0.16,0.22)					0.05 (-0.11,0.20)			**0.29 (0.07,0.53)**			**MMDHP+CD**								
**0.91 (0.76,1.05)**		0.10 (-0.23,0.44)	0.09 (-0.12,0.28)					0.10 (-0.07,0.26)			**0.35 (0.11,0.58)**			0.05 (-0.15,0.24)		**SDMUC+CD**						
(F) Network meta-analysis comparisons for macular thickness																						
**CD**																						
**68.87 (36.48,101.20)**		**CDDP+CD**																				
**70.76 (55.83,85.51)**		1.88 (-33.73,37.47)	**CXC+CD**																			
45.19 (-0.48,90.77)		-23.67 (-79.56,32.22)	-25.57 (-73.48,22.60)					**HXMMT+CD**														
**52.16 (19.76,84.49)**		-16.71 (-62.45,29.08)	-18.60 (-54.01,17.02)					6.98 (-49.01,62.74)			**MMDHP+CD**											
22.01 (-23.93,67.98)		-46.85 (-102.96,9.49)	-48.75 (-97.01,-0.34)					-23.23 (-87.96,41.40)			-30.15 (-86.36,26.06)			**QG**								
**47.06 (14.64,79.50)**		-21.85 (-67.65,24.15)	-23.69 (-59.28,12.00)					1.85 (-54.22,57.84)			-5.09 (-50.87,40.55)			25.04 (-31.22,81.28)			**SDMUC+CD**					

The differences between the compared groups were deemed as significant when the 95% CrI of the OR did not contain 1.00 or the MD did not contain 0.00, which is marked as bold font. The data are the OR (95% CrI) of the column intervention compared to the row intervention, i.e., for the clinical effective rate, CD alone was significantly less effective than CDDP alone (OR 0.16, 95% CrI 0.03,0.69). CD, calcium dobesilate; CXC, Compound Xueshuantong Capsule; CDDP, Compound Danshen Dripping Pill; SDMUC, Shuangdan Mingmu Capsule; QG, Qiming Granule; HXMMT, Hexuemingmu Tablet; DHHYK, Danhong Huayu Koufuye; MMDHP, Mingmu Dihuang Pill.

**Table 3:** Ranking probability of interventions.

Intervention	Clinical effective rate		Visual acuity		Visual field gray value		Microaneurysm volume		Hemorrhage area		Macular thickness	
	SUCRA (%)	Rank	SUCRA (%)	Rank	SUCRA (%)	Rank	SUCRA (%)	Rank	SUCRA (%)	Rank	SUCRA (%)	Rank
CXC+CD	54.02	6	98.51	1	56.74	4	73.19	2	55.29	5	86.23	1
CDDP+CD	88.58	1	41.8	4	59.74	3	36.45	4	59.2	3	80.94	2
QG+CD	66.98	3	49.27	3	–	–	–	–	–	–	–	–
SDMUC+CD	60.63	4	–	–	–	–	–	–	86.24	1	49.94	4
MMDHP+CD	43.56	8	–	–	67.47	2	45.87	3	72.29	2	57.05	3
HXMMT+CD	40.01	9	–	–	23.7	5	94.48	1	18.67	6	48.82	5
CXC	39.97	10	–	–	–	–	–	–	–	–	–	–
CDDP	69.27	2	70.34	2	91.83	1	–	–	58.3	4	–	–
QG	57.18	5	–	–	–	–	–	–	–	–	23.91	6
SDMUC	47.47	7	–	–	–	–	–	–	–	–	–	–
DHHYK	30.04	11	33.85	5	–	–	–	–	–	–	–	–
CD	2.27	12	6.25	6	0.51	6	0	5	0	7	3.11	7

CD, calcium dobesilate; CXC, Compound Xueshuantong Capsule; CDDP, Compound Danshen Dripping Pill; SDMUC, Shuangdan Mingmu Capsule; QG, Qiming Granule; HXMMT, Hexuemingmu Tablet; DHHYK, Danhong Huayu Koufuye; MMDHP, Mingmu Dihuang Pill, -: no value.

#### 3.5.2 Visual acuity

Eight studies informed the improvement of visual acuity. CXC+CD obtained a better effect than CDDP+CD, QG+CD, DHHYK, and CD alone (**Table 2B**). Based on the ranking probability of SUCRA, CXC+CD ranked first (98.51%), followed by CDDP (70.34%) and QG+CD (49.27%), whereas CD alone obtained the worst effect (6.25%). The detail is shown in **Figure 4B** and **Table 3**.

#### 3.5.3 Visual field gray value

The assessment of the visual field gray value included six interventions. Four interventions (CDDP, CDDP+CD, CXC+CD, and MMDHP+CD) could improve the visual field gray value compared to CD alone (**Table 2C**). According to the ranking probability of SUCRA, CDDP had the highest SUCRA value (91.83%), followed by MMDHP+CD (67.47%) and CDDP+CD (59.74%), whereas CD alone obtained the worst effect (0.51%). The detail is shown in **Figure 4C** and **Table 3**.

#### 3.5.4 Microaneurysm volume

Fourteen studies involving five interventions reported microaneurysm volume. Four treatment types (CDDP+CD, CXC+CD, HXMMT+CD, and MMDHP+CD) showed the ability to reduce microaneurysm volume more than CD alone, while other treatments did not show significant differences. Based on the ranking probability of SUCRA, HXMMT+CD might have the highest possibility (94.48%), while CD alone might be the least improved treatment (0.0%). The detail is shown in **Figure 4D** and **Table 3**.

#### 3.5.5 Hemorrhage area

Eighteen studies informed the decrease of hemorrhage area. We found that all the included OCPMs, including CDDP, CDDP+CD, CXC+CD, HXMMT+CD, MMDHP+CD, and SDMUC+CD, reduced hemorrhage area more than CD alone (**Table 2E**). Based on the ranking probability of SUCRA, SDMUC+CD ranked first (86.24%), followed by MMDHP+CD (72.29%) and CDDP+CD (59.2%), whereas CD alone obtained the worst effect (0.0%). The detail is shown in **Figure 4E** and **Table 3**.

#### 3.5.6 Macular thickness

Eighteen studies involving 7 interventions reported macular thickness. Network comparisons suggested that four treatment types (CDDP+CD, CXC+CD, MMDHP+CD, and SDMUC+CD) were better than CD alone in reducing macular thickness (**Table 2F**). According to SUCRA, CXC+CD had the highest SUCRA value (86.23%), followed by CDDP+CD (80.94%) and MMDHP+CD (57.05%), whereas CD alone obtained the worst effect (3.11%). The detail is shown in **Figure 4F**, and **Table 3**.

#### 3.5.7 Adverse drug reactions

In this study, The NMA for ADRs was difficult because 8 of the 22 included studies reported no adverse reactions in the experimental and control groups. The other 14 studies reported 160 cases of adverse drug reactions, including 2 studies that did not identify specific ADRs. According to the results of the pairwise meta-analysis, there were no adverse reactions in the experimental and control groups, apart from QG, in which those of the OCPMs were lower than those of CD alone (**Supplementary File S5**).

### 3.6 Sensitivity analysis

For sensitivity analysis, we excluded RCTs with high risk of bias (2 studies) and RCTs with short treatment courses of 1-2 months (6 studies), respectively. The NMA results were re-evaluated. We found that both the effect size and direction did not change significantly, only the confidence intervals have gotten a little bit wider. The ranking probabilities were also not changed substantially. These suggested that the NMA results are robust to a certain extent. The details are shown in **Supplementary Files S8**.

### 3.7 Publication bias

**Figure 5:**
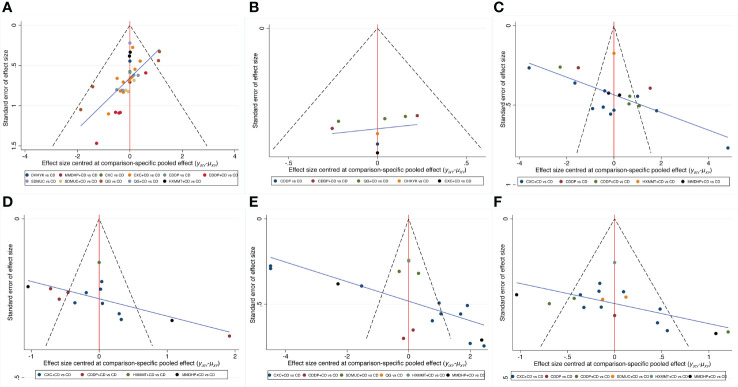
Comparison-adjusted funnel plot for six outcomes. **(A)** Clinical effective rate; **(B)** Visual acuity; **(C)** Visual field gray value; **(D)** Microaneurysm volume; **(E)** Hemorrhage area; **(F)** Macular thickness. CD, calcium dobesilate; CXC, Compound Xueshuantong Capsule; CDDP, Compound Danshen Dripping Pill; SDMUC, Shuangdan Mingmu Capsule; QG, Qiming Granule; HXMMT, Hexuemingmu Tablet; DHHYK, Danhong Huayu Koufuye; MMDHP, Mingmu Dihuang Pill.

**Figure 5** depicts the comparison-adjusted funnel plot for six outcomes to assess publication bias. It can be seen that the calibration auxiliary line was not completely perpendicular to the centerline, suggesting that the NMA may have potential publication bias.

## 4 Discussion

In recent decades, diabetes has progressed from a disease occurring mainly in populations of developed countries to a worldwide epidemic. DR, which is a major cause of visual impairment in a continuously increasing number of diabetic patients, is a common complication of diabetes (4). Once patients reach the PDR stage, they may progress to more serious diseases such as retinal neovascularization, vitreous hemorrhage, and retinal detachment, which can severely disrupt the visual function of patients even with interventions such as laser and vitrectomy (60). Therefore, early treatment and prevention at the stage of NPDR have become an urgent clinical problem to be solved. In China, clinicians often apply OCPMs for the treatment of NPDR with good clinical efficacy (11). DR belongs to the category of “thirsty eye disease” according to traditional Chinese medicine, and its pathogenesis is mainly attributed to the deficiency of both qi and yin and the stasis of ocular collateral (61). Based on this etiology and pathogenesis, the clinical treatment of DR can be based on Chinese herbal medicines that benefit Qi, nourish Yin, invigorate blood, and disperse blood stasis, and the OCMPs selected for this NMA all belong to this category.

### 4.1 Main findings

This NMA systematically evaluated the efficacy and safety of 7 commonly used OCPMs (CXC, CDDP, QG, SDMUC, MMDHP, HXMMT, and DHHYK) alone or combined with CD in patients with NPDR based on information from 42 studies involving 7 categories of outcomes. Our results showed that OCPMs alone or combined with CD were superior to CD alone in terms of an improved clinical efficacy rate, improved visual acuity and visual field gray value, reduced fundus hemorrhage and exudation, and better drug safety, which is consistent with the results of a published pairwise meta-analysis (11). More so, by comparing different types of proprietary Chinese medicines, the NMA also suggests that more attention should be given to CDDP, CXC, HXMMT, and SDMMC in the clinical treatment of NPDR.

The NMA results suggested that CDDP+CD and CDDP alone might be the best treatment options for increasing clinical efficacy rate and improving visual field gray value. CDDP is a herbal compound used for the treatment of cardiovascular diseases, made from *Salvia miltiorrhiza* Bunge [Lamiaceae], *Panax notoginseng* (Burkill) F.H.Chen [Araliaceae] and a small number of Borneolum Syntheticum, and contain active ingredients such as tanshinone, protocatechuic acid, ginsenoside, and Panax ginsenoside (62). Due to its vascular protective effects (63), CDDP is often used clinically in patients with DR. A Meta-analysis that included eight RCTs involving 524 patients found that CDDP combined with western drugs was effective in improving patients’ visual function (64), which is consistent with our present NMA results. Meanwhile, basic studies have shown that CDDP can improve retinal vascular and neurological function by inhibiting inflammation, oxidative stress, and apoptosis, reducing vascular endothelial cell damage, and increasing retinal thickness (65, 66), which may explain the superiority of CDDP over other OCPMs in improving visual function.

We found that CXC+CD might be the best choice for improving visual acuity and reducing macular thickness. CXC is composed of four herbal medicines, *Panax notoginseng* (Burkill) F.H.Chen [Araliaceae], *Astragalus mongholicus* Bunge [Fabaceae], *Salvia miltiorrhiza* Bunge [Lamiaceae], and *Scrophularia ningpoensis* Hemsl. [Scrophulariaceae], and its active ingredients are mainly quercetin, luteolin, kaempferol, and tanshinone Iia, which may be related to the tumor necrosis factor (TNF) signal pathway, hypoxia-inducible factor-1 signal pathway, and vascular endothelial growth factor (VEGF) signal pathway in DR (67). Modern pharmacological studies have shown that CXC can reduce retinal damage by reducing erythrocyte aggregation and lowering plasma viscosity, as well as inhibiting aldose reductase activity, controlling high expression of VEGF, and intercellular cell adhesion molecule-1 (68, 69). In addition, research has also revealed that CXC protects against high glucose-damaged retinal vascular endothelial cells, thereby reducing the blood-retinal intra-retinal barrier damage and decreasing retinal leakage (9). Compared with other OCPMs, CXC contains *Astragalus mongholicus* Bunge [Fabaceae], which could replenish Qi, lift Yang, promote water, and reduce swelling. These may explain why CXC is superior to other OCPMs in improving visual acuity and reducing macular thickness.

The NMA showed that HXMMT+CD had the best effect of reducing the microaneurysm volume. HXMMT is composed of 18 herbs. Modern pharmacological studies have shown that HXMMT can effectively inhibit platelet aggregation and adhesion and improve blood rheology (70, 71). In addition, several clinical studies have shown that HXMMT can effectively improve fundus hemorrhage and exudation, and stabilize lesion efficacy in patients with NPDR (72, 73). *In vitro* experiments, HXMMT can effectively reduce retinal damage through antioxidant, anti-inflammatory, and anti-angiogenic properties (74). While there are fewer *in vivo* studies on HXMMT in DR, they only study applied HXMMT to rats with branch retinal vein obstruction and found that it could improve retinal edema and enhance retinal function by improving retinal microcirculation and regulating VEGF-α expression (75). Compared with other OCPMs, HXMMT contains Typha angustifolia L and salvia, which are commonly used clinically to stop bleeding and resolve blood stasis. Meanwhile, HXMMT has more therapeutic targets shared by different active ingredients and more signaling pathways, such as neuroactive ligand-receptor interaction and chemokine signaling pathway (71), which may be the reason why HXMMT has the best efficacy in reducing the microaneurysm volume.

SDMMC+CD may be the best intervention in reducing retinal hemorrhage area, which is consistent with the previously existing pairwise Meta-analysis results (76). Published studies have confirmed that SDMMC can improve glucose dyslipidemia and blood rheology, reduce oxidative stress, and can effectively improve fundus signs, protect the retinal structure, and reduce retinal hemorrhage in patients with DR (77, 78). Experimental studies have shown that SDMMC can improve the blood rheological status of DR rats, dilate retinal arteries, and improve retinal blood supply (79). Compared with other OCPMs, SDMMC contains more blood-cooling herbs such as *Ligustrum lucidum* W.T.Aiton [Oleaceae], *Eclipta prostrata* (L.) L. [Asteraceae], has been proven to reduce the inflammatory response and inhibit neovascularization by decreasing the expression of VEGFA and TNF-α (80). It may explain the better effect of SDMMC in reducing the retinal hemorrhage area than other OCPMs.

ADRs also deserve our attention apart from efficacy. However, the NMA showed that the OCPMs selected in this study did not cause serious adverse reactions. To use OCPMs in a manner that will limit ADRs, we suggest that clinicians applying OCPMs should give different types of OCPMs according to the actual situation of the patients and patients should avoid taking OCPMs on an empty stomach.

### 4.2 Strengths and limitations

To our knowledge, this is the first comprehensive evaluation of different types of OCPMs alone or in combination with CD for NPDR using a reticulated meta-analysis, and the recommended ranking order based on efficacy and safety provides a usable basis for clinicians. Our study has the following strengths: (1) The screening criteria were strict, with the study population limited to patients with NPDR and CD as a fixed control, which ensured the uniformity of disease and interventions, thus reducing heterogeneity to some extent; (2) The OCPMs selected are those recommended in the NHSA and NMPA catalogs to ensure consistency with the actual clinic. (3) This NMA not only focuses on efficacy indicators such as the clinical efficiency rate, visual acuity, visual field gray value, microaneurysm volume, hemorrhage area, and macular thickness, but also the incidence of ADRs; (4) This NMA projects the optimal treatment for each outcome indicator according to SUCRA, which can be used as a reference for the clinic.

Although the current NMA may fill the gap in the efficacy of different types of OCPMs for NPDR, there are still some limitations: (1) The overall risk of the vast majority of studies was rated as “some concern”. And the criteria for clinical efficacy of included studies were not completely consistent, though all studies were based on the improvement of visual acuity and fund signs, so the NMA results should be interpreted cautiously; (2) The number of studies included in some interventions was limited, with only two, one, and two RCTs that included HXMMT, DHHYK, and MMDHP, respectively; (3) The studies included in the current NMA are all from China, so the NMA results may not apply to other countries; (4) Some of the OCPMs contain the same monomers which may exert similar therapeutic effects and may not fully explain the conclusions reached in our study. The current data extracted from clinical trials are incapable of exploring interactive effects of integrative therapies and combination of multiple herbal substances, further studies focusing on pharmacological features of OCPMs are needed.

### 4.3 Implications for future RCTs of OCPMs

A large number of RCTs on OCPMs have been published, but issues regarding poor reporting and the high potential of bias have attracted widespread attention (81). We believe that the reasons for the poor quality of RCTs on OCPMs may include the following: (1) In terms of policy, the regulatory system for OCPMs is not robust enough. Reports of phase I, II, and III trials for OCPMs are hardly available in public; (2) Most RCTs for OCPMs do not have pre-specified study protocols and prospective registration, and thus lack detailed information on sample size calculation, randomization procedure, blinding, etc. (3) Most of the trials did not follow RCT reporting guidelines such as SPIRIT and CONSORT (82, 83); (4) Although these OPCMs are currently in regular clinical use in China, most of the published RCTs are single-center studies with an early publication year and lack data updates (84).

In order to enhance the reporting and conducting of RCTs for OCPMs, the following four efforts are needed: (1) The regulatory system for Chinese patent medicines should be strengthened, and publication of trial results conducted by pharmaceutical companies and relevant affiliations should be encouraged by the authority to improve transparency; (2) Investigators should develop detailed study protocols in advance and register their trial in the clinical trial registration platform (https://www.chictr.org.cn/index.aspx); (3) Protocol of RCTs should be reported according to SPIRIT and final report should be reported according to the CONSORT statement. (4) Multicenter and large-sample RCTs for OCPMs are recommended. Moreover, future research should pay more attention on key methodological issues and the quality of conducting.

## 5 Conclusions

This NMA evaluated the efficacy and safety of OCMPs alone or combined with CD for the treatment of patients with NPDR. The results showed that OCMPs combined with CD or alone were efficient in improving visual function and fundus signs in NPDR patients. In terms of improving the clinical efficacy rate and visual field gray value, CDDP combined with CD and CDDP alone may be the best intervention. Regarding improving visual acuity and reducing macular thickness, CXC combined with CD may be the most effective treatment option. In terms of reducing microaneurysm volume and hemorrhage area, HXMMT and SDMMC combined with CD may be the most effective. The OCPMs could increase clinical efficacy, and neither had a significantly increased risk of ADRs. However, regarding the limitations in methodology and potential risk of bias, more RCTs with high quality are needed to confirm the evidence of the NMA results. OCPMs should be used with more caution in clinical practice. Besides, RCTs of OCPMs should pay more attention on key methodological issues and the quality of conducting in the future.

## Data availability statement

The original contributions presented in the study are included in the article/**Supplementary Material**. Further inquiries can be directed to the corresponding author.

## Author contributions

ZL and YC: conceptualization, methodology, formal analysis, and writing the original draft. YD and XH: methodology and supervision. YL and WC: visualization and review editing. JW: language editing and supervision. CJ: conceptualization, funding, and project administration. All authors contributed to the article and approved the submitted version.

## References

[1] Sun H Saeedi P Karuranga S Pinkepank M Ogurtsova K Duncan BB . IDF diabetes atlas: Global, regional and country-level diabetes prevalence estimates for 2021 and projections for 2045. Diabetes Res Clin Pract (2022) 183:109119. doi: 10.1016/j.diabres.2021.109119 34879977 PMC11057359

[2] Li Y Teng D Shi X Qin G Qin Y Quan H . Prevalence of diabetes recorded in mainland China using 2018 diagnostic criteria from the American diabetes association: national cross sectional study. BMJ (Clinical Res ed.) (2020) 369:m997. doi: 10.1136/bmj.m997 PMC7186854 32345662

[3] Wilkinson CP Ferris FL Klein RE Lee PP Agardh CD Davis M . Proposed international clinical diabetic retinopathy and diabetic macular edema disease severity scales. Ophthalmology (2003) 110(9):1677–82. doi: 10.1016/S0161-6420(03)00475-5 13129861

[4] Cheung N Mitchell P Wong TY . Diabetic retinopathy. Lancet (London England) (2010) 376(9735):124–36. doi: 10.1016/S0140-6736(09)62124-3 20580421

[5] Zhang X Liu W Wu S Jin J Li W Wang N . Calcium dobesilate for diabetic retinopathy: A systematic review and meta-analysis. Sci China Life Sci (2015) 58(1):101–7. doi: 10.1007/s11427-014-4792-1 25528255

[6] Liu J Li S Sun D . Calcium dobesilate and micro-vascular diseases. Life Sci (2019) 221:348–53. doi: 10.1016/j.lfs.2019.02.023 30769115

[7] Stamper RL Smith ME Aronson SB Cavender JC Cleasby GW Fung WE . The effect of calcium dobesilate on nonproliferative diabetic retinopathy: a controlled study. Ophthalmology (1978) 85(6):594–606. doi: 10.1016/s0161-6420(78)35643-8 353623

[8] Jia Y Sun J Zhao Y Tang K Zhu R Zhao W . Chinese Patent medicine for osteoporosis: A systematic review and meta-analysis. Bioengineered (2022) 13(3):5581–97. doi: 10.1080/21655979.2022.2038941 PMC8973707 35184684

[9] Xing W Song Y Li H Wang Z Wu Y Li C . Fufang xueshuantong protects retinal vascular endothelial cells from high glucose by targeting YAP. Biomed. Pharmacother. = Biomed. Pharmacotherapie (2019) 120:109470. doi: 10.1016/j.biopha.2019.109470 31590124

[10] Nie F Yan J Ling Y Liu Z Fu C Li X . Effect of shuangdan mingmu capsule, a Chinese herbal formula, on oxidative stress-induced apoptosis of pericytes through PARP/GAPDH pathway. BMC Complementary Med Therapies (2021) 21(1):118. doi: 10.1186/s12906-021-03238-w PMC8037833 33838689

[11] Zhang Y An X Duan L Jin D Duan Y Zhou R . Effect of Chinese patent medicines on ocular fundus signs and vision in calcium dobesilate-treated persons with non-proliferative diabetic retinopathy: A systematic review and meta-analysis. Front Endocrinol (2022) 13:799337. doi: 10.3389/fendo.2022.799337 PMC8967137 35370950

[12] Hutton B Salanti G Caldwell DM Chaimani A Schmid CH Cameron C . The PRISMA extension statement for reporting of systematic reviews incorporating network meta-analyses of health care interventions: checklist and explanations. Ann Internal Med (2015) 162(11):777–84. doi: 10.7326/M14-2385 26030634

[13] Fundus Disease Group, O.S.o.C.M.A . Guidelines for clinical diagnosis and treatment of diabetic retinopathy, (2014). Chin J Ophthalmol (2014) 50(11):851–65. doi: 10.3760/cma.j.issn.0412-4081.2014.11.014

[14] Cumpston M Li T Page MJ Chandler J Welch VA Higgins JP . Updated guidance for trusted systematic reviews: A new edition of the cochrane handbook for systematic reviews of interventions. Cochrane Database Systematic Rev (2019) 10:ED000142. doi: 10.1002/14651858.ED000142 PMC10284251 31643080

[15] Béliveau AA-O Boyne DJ Slater J Brenner D Arora P . BUGSnet: an r package to facilitate the conduct and reporting of Bayesian network meta-analyses. BMC Med Res Methodol (2019) 19:196. doi: 10.1186/s12874-019-0829-2 31640567 PMC6805536

[16] Salanti G . Indirect and mixed-treatment comparison, network, or multiple-treatments meta-analysis: Many names, many benefits, many concerns for the next generation evidence synthesis tool. Res synthesis Methods (2012) 3(2):80–97. doi: 10.1002/jrsm.1037 26062083

[17] Dias S Sutton AJ Ades AE Welton NJ . Evidence synthesis for decision making 2: a generalized linear modeling framework for pairwise and network meta-analysis of randomized controlled trials. Med Decis Making (2013) 33(5):607–17. doi: 10.1177/0272989X12458724 PMC3704203 23104435

[18] Du JH Zhang Z Li JQ Zhu XR Chai XH Gong L . Effect of compound xueshuantong on blood lipid and fundus microaneurysm in diabetic retinopathy. Modern J Integrated Traditional Chin Western Med (2018) 27(06):663–5. doi: CNKI:SUN:XDJH.0.2018-06-030

[19] Huang W Tang XD Liu J . Analysis of the therapeutic effect of calcium dobesilate combined with compound xueshuantong capsules on diabetic retinopathy. Contemp Med (2021) 27(31):162–4. doi: 10.3969/j.issn.1009-4393.2021.31.066

[20] An LN . Clinical study of compound xueshuantong combined with calcium dobesilate in the treatment of diabetic retinopathy. Pract Clin J Integrated Traditional Chin Western Med (2020) 20(14):69–71. doi: 10.13638/j.issn.1671-4040.2020.14.034

[21] Wang J Du W Li Y . Effect of calcium oxybenzenesulfate combined with compound xueshuantong capsule on senile diabetic retinopathy and hemorheology. Chin J Gerontol. (2020) 40(08):1603–6. doi: 10.3969/j.issn.1005-9202.2020.08.010

[22] Yan H Song JH . Effect of compound xueshuantong capsule in the adjuvant treatment of non proliferative diabetic retinopathy and its influence on the hemodynamics of patients with OA. Strait Pharm J (2020) 32(05):141–2. doi: 10.3969/j.issn.1006-3765.2020.05.060

[23] Chai F Wang Y Huang Q . Effects of compound xueshuantong capsule combined with calcium dobesilate on IGF-1, VEGF and hemodynamic indexes in early diabetic retinopathy. J Pract Traditional Chin Med (2018) 34(11):1360–2. doi: 10.3969/j.issn.1004-2814.2018.11.068

[24] Wang Q . Clinical efficacy of compound xueshuantong capsule combined with calcium oxybenesulfate in the treatment of early diabetic retinopathy and its influence on hs-CRP and IGF-1 levels. Pract Clin J Integrated Traditional Chin Western Med (2018) 18(06):16–17+53. doi: 10.13638/j.issn.1671-4040.2018.06.008

[25] Ma JP . Curative effect evaluation of compound xueshuantong capsule combined with calcium dobesilate for patients with early diabetic retinopathy. Int Eye Sci (2018) 18(02):305–8. doi: 10.3980/j.issn.1672-5123.2018.2.25

[26] Yu W Lin BS Ma SF Zhang WW Wu QL . Effect of calcium oxybenesulfate combined with compound xueshuantong capsule on early diabetic retinopathy and serum IGF-1 and VEGF levels. Chin J Gerontol. (2017) 37(21):5311–3. doi: 10.3969/j.issn.1005-9202.2017.21.041

[27] Rao XJ Wu YM Wei LJ Ma Y . Comparison of efficacy between traditional Chinese medicine combined with western medicine and simple western medicine for patients with NPDR. Int Eye Sci (2017) 17(01):148–50. doi: 10.3980/j.issn.1672-5123.2017.1.41

[28] Men LB . Effects of relapse-xueshuantong combined with calcium oxybenesulfate on visual acuity and levels of inflammatory factors in patients with diabetic retinopathy. Clin Med (2020) 40(01):124–5. doi: 10.19528/j.issn.1003-3548.2020.01.051

[29] Li Y . Effects of compound xueshuantong combined with calcium oxybenesulfate on vascular growth factor levels and visual acuity in patients with diabetic retinopathy. Diabetes New World (2019) 22(07):180–1. doi: 10.16658/j.cnki.1672-4062.2019.07.180

[30] Pei D Gao H . Clinical effects and hs-CRP,VEGF and IGF-1 levels of xueshuantong capsule combined with calcium dobesilate in treatment of early diabetic retinopathy. Modern J Integrated Traditional Chin Western Med (2015) 24(35):3896–3898,3907. doi: 10.3969/j.issn.1008-8849.2015.35.007

[31] Zhou YD Zheng MR Wang FF . Clinical studv of calcium dobesilate combined with compound xueshuantong in the treatment of diabetic retinopathy. Sichuan J Anat (2022) 30(1):62–4. doi: 10.3969/j.issn.1005-1457.2022.01.021

[32] Luo D Qin Y Yuan W Deng H Zhang Y Jin M . Compound danshen dripping pill for treating early diabetic retinopathy: A randomized, double-dummy, double-blind study. Evidence-Based Complementary Altern Med (2015) 2015:1–7. doi: 10.1155/2015/539185 PMC4592726 26457110

[33] Jin M Deng H Yuan W Yang G . Clinical observation of compound salvia miltiorrhiza dripping pills in the treatment of early diabetic retinopathy. Chin Community Doctors (2009) 25(16):32–3. doi: CNKI:SUN:XCYS.0.2009-16-033

[34] Chen Y Zhong JY . Clinical study of compound salvia miltiorrhiza dripping pills in the treatment of simple diabetic retinopathy. Chin Community Doctors (2006) 23):75. doi: 10.3969/j.issn.1672-5085.2011.35.124

[35] Xu HT . Effect of compound salvia miltiorrhiza dripping pills combined with calcium oxybenzenesulfate in the treatment of diabetic retinopathy. Chin J Convalescent Med (2019) 28(08):884–6. doi: 10.13517/j.cnki.ccm.2019.08.041

[36] Li Y . Clinical efficacy evaluation of the combination regimen of calcium oxybenesulfate capsules and compound danshen dripping pills in the treatment of diabetic retinopathy. J Aerospace Med (2017) 28(10):1229–31. doi: 10.3969/j.issn.2095-1434.2017.10.037

[37] Wang HM Tian DZ Han XY . Effect of compound salvia miltiorrhiza dripping pills on non-proliferative diabetic retinopathy. Chin J Clin Rational Drug Use (2016) 9(23):49–50. doi: 10.15887/j.cnki.13-1389/r.2016.23.035

[38] Bai YX . Effect of calcium dobesilate combined with compound danshen dripping pills on diabetic retinopathy and serum inflammatory factors. J Qiqihar Med Univ (2017) 38(22):2641–3. doi: 10.3969/j.issn.1002-1256.2017.22.014

[39] Ruan YX Chen M Liu ZQ Wang ZL Sun N Huang X . Clinical study on the treatment of diabetic retinopathy by oral compoundDanshen dripping pill combined with calcium dobesilate. Med Sci J Cent South China (2017) 45(01):18–20+23. doi: 10.15972/j.cnki.43-1509/r.2017.01.004

[40] Huang YX Sun HP Tang YQ Chen X . Effects of compound danshen dripping pills on inflammatory mediators, cytokines and visual function in patients with diabetic retinopathy. Chin J Integr Med Cardio-Cerebrovascular Dis (2021) 19(08):1364–1366+1408. doi: 10.12102/j.issn.1672-1349.2021.08.030

[41] Qin YH Li F Tu LY Qiu B Zhang ML Cao JH . Multicentric clinical study of shuangdan mingmu capsule on diabetic retinopathy. J Hunan Univ Chin Med (2010) 1):46–51. doi: 10.3969/j.issn.1674-070X.2010.01.015

[42] Ji XD Liu WT . Safety and efficacy of shuangdan mingmu capsule combined with calcium dobesilate dispersible tablets in the treatment of diabetic retinopathy. Clin Res Pract (2022) 7(11):85–7. doi: 10.19347/j.cnki.2096-1413.202211024

[43] Liu JP Kong YH Wang L . Clinical efficacy of shuangdan mingmu capsules combined with calcium dobesilate in the treatment of diabetic retinopathy and its effects on serum levels of vascular endothelial growth factor, platelet-derived growth factor and interleukin-1. Eval Anal Drug-Use Hospitals (2019) 19(11):1332–1334+1338. doi: 10.14009/j.issn.1672-2124.2019.11.015

[44] Jin L Zhang LJ . Clinical study on shuangdan mingmu capsules combined with calcium dobesilate in treatment of diabetic retinopathy. Drugs Clinic (2019) 34(01):159–63. doi: 10.7501/j.issn.1674-5515.2019.01.035

[45] Pang YH . Clinical observation of shuangdanmingmu capsule in treating diabetic retinopathy. Diabetes New World (2015) 35(3):39–9. doi: 10.16658/j.cnki.1672-4062.2015.03.013

[46] Zhang DX . Randomized controlled study of qiming granule and dishengming capsule in the treatment of diabetic retinopathy. Pharmacol Clinics Chin Materia Med (2015) 31(3):151–2. doi: 10.13412/j.cnki.zyyl.2015.03.050

[47] Fang J Lv H Zhang XD Liu F Yang LN . Clinical study on qiming granules for non-proliferative diabetic retinopathy. New Chin Med (2022) 54(08):97–9. doi: 10.13457/j.cnki.jncm.2022.08.022

[48] Duan JG Liao PZ Wu L Li SM Yu YG Qiu B . Randomized controlled double-blind multicentric clinical trail on non-proliferative DiabeticRetinopathy treated by qi-ming granule. J Chengdu Univ Traditional Chin Med (2006) 29(2):1–5. doi: CNKI:SUN:CDZY.0.2006-02-000

[49] Fan YP Li YL Chen GL Wang HH Lin YJ . Effect and safety evaluation of qiming granule on the treatment of nonproliferative diabetic retinonathy. Int Eye Sci (2018) 18(12):2260–3. doi: 10.3980/j.issn.1672-5123.2018.12.34

[50] Feng JL Zhou P Wu QL Chen YN Tian J . Effect of qiming granule combined with calcium dobesilate on diabetic retinopathy in the third stage. Hebei Med J (2016) 38(22):3430–3. doi: 10.3969/j.issn.1002-7386.2016.22.018

[51] Wang ZQ Lei Y Zhang R . Improvement of qiming granule combined with calcium dobesilate on fundus microcirculation in the treatment of patients with non-proliferative diabetic retinopathy. Clin Res Pract (2019) 4(31):117–8. doi: 10.19347/j.cnki.2096-1413.201931049

[52] Wang ZZ . Qi Ming granule combined with calcium dobesilate in treatment of nonproliferative diabetic retinopathy. Int Eye Sci (2017) 17(04):702–5. doi: 10.3980/j.issn.1672-5123.2017.4.28

[53] Sui HL Yu CY Xue HM Wang RN . The clinical curative observation on qiming granule combined with calcium dobesilate capsules in treatment of patients with nonproliferative diabetic retinopathy. Med Innovation China (2014) 11(20):99–102. doi: 10.3969/j.issn.1674-4985.2014.20.036

[54] Yan JH . Effect of qiming granule combined with calcium dobesilate in the treatment of non-proliferative diabetic retinopathy. Henan Med Res (2020) 29(34):6477–9. doi: 10.3969/j.issn.1004-437X.2020.34.055

[55] Ye XL Guo XN Xiong F . Clinical observation of oral calcium dobesilate combined with HeXueMingMu tablet in the treatment of nonproliferative diabetic retinopathy. J North Sichuan Med Coll (2019) 34(2):223–5. doi: 10.3969/j.issn.1005-3697.2019.02.16

[56] Gao L Qi T Xu WB Wan L . Hexuemingmu tablets combined with calcium dobesilate on early DiabeticRetinopathy. China Pharm (2020) 29(10):133–5. doi: 10.3969/j.issn.1006-4931.2020.10.040

[57] Zhu HM Jiang Y Li L Yu S . Efficacy of danhong huayu in treatment of diabetic retinopathy. Chin J Exp Traditional Med Formulae (2013) 19(17):320–3. doi: 10.11653/syfj2013170320

[58] Li JB Wei XY . Clinical study on mingmu dihuang pills combined with calcium dobesilate in treatment of early diabetic retinopathy. Drugs Clinic (2019) 34(04):1197–201. doi: CNKI:SUN:GWZW.0.2019-04-069

[59] Ayinu-Nulahou Wang YH Bu Q Zhao Y . Clinical efficacy of calcium dobesilate dispersible tablets combined withMingmu dihuang pills in treatment of NPDR. Int Eye Sci (2019) 19(6):992–6. doi: 10.3980/j.issn.1672-5123.2019.6.23

[60] Kollias AN Ulbig MW . Diabetic retinopathy: Early diagnosis and effective treatment. Deutsches Arzteblatt Int (2010) 107(5):75–83; quiz 84. doi: 10.3238/arztebl.2010.0075 PMC2828250 20186318

[61] Jie CH . Constructing the thinking of syndrome differentiation and treatment of diabetic retinopathy under the mode of combination of disease-syndrome symptom. China J Chin Ophthalmol (2022) 32(11):841–5. doi: 10.13444/j.cnki.zgzyykzz.2022.11.001

[62] Liao W Ma X Li J Li X Guo Z Zhou S . A review of the mechanism of action of dantonic^®^ for the treatment of chronic stable angina. Biomed. Pharmacother. = Biomed. Pharmacotherapie (2019) 109:690–700. doi: 10.1016/j.biopha.2018.10.013 30551521

[63] Hu Y Sun J Wang T Wang H Zhao C Wang W . Compound danshen dripping pill inhibits high altitude-induced hypoxic damage by suppressing oxidative stress and inflammatory responses. Pharm Biol (2021) 59(1):1585–93. doi: 10.1080/13880209.2021.1998139 PMC8635678 34808069

[64] Huang H Li Y Huang Q Lei R Zou W Zheng Y . Efficacy of compound danshen dripping pills combined with western medicine in the treatment of diabetic retinopathy: a systematic review and meta-analysis of randomized controlled trials. Ann Palliative Med (2021) 10(10):10954–62. doi: 10.21037/apm-21-2563 34763458

[65] Zhang Q Xiao X Zheng J Li M Yu M Ping F . Compound danshen dripping pill inhibits retina cell apoptosis in diabetic rats. Front Physiol (2018) 9:1501. doi: 10.3389/fphys.2018.01501 30405447 PMC6207599

[66] Liu L Li X Cai W Guo K Shi X Tan L . Coadministration of compound danshen dripping pills and bezafibrate has a protective effect against diabetic retinopathy. Front Pharmacol (2022) 13:1014991. doi: 10.3389/fphar.2022.1014991 36278163 PMC9579276

[67] Li H Li B Zheng Y . Exploring the mechanism of action compound-xueshuantong capsule in diabetic retinopathy treatment based on network pharmacology. Evidence-Based Complementary Altern Medicine: eCAM (2020) 2020:8467046. doi: 10.1155/2020/8467046 PMC7499338 32963574

[68] Jian W Yu S Tang M Duan H Huang J . A combination of the main constituents of fufang xueshuantong capsules shows protective effects against streptozotocin-induced retinal lesions in rats. J Ethnopharmacol (2016) 182:50–6. doi: 10.1016/j.jep.2015.11.021 26692279

[69] Hao GM Lv TT Wu Y Wang HL Xing W Wang Y . The hippo signaling pathway: A potential therapeutic target is reversed by a Chinese patent drug in rats with diabetic retinopathy. BMC Complementary Altern Med (2017) 17(1):187. doi: 10.1186/s12906-017-1678-3 PMC5379696 28372586

[70] Fei YX Wang SQ Yang LJ Qiu YY Li YZ Liu WY . Salvia miltiorrhiza bunge (Danshen) extract attenuates permanent cerebral ischemia through inhibiting platelet activation in rats. J Ethnopharmacol. (2017) 207:57–66. doi: 10.1016/j.jep.2017.06.023 28645780

[71] Yao H Xin D Zhan Z Li Z . Network pharmacology-based approach to comparatively predict the active ingredients and molecular targets of compound xueshuantong capsule and hexuemingmu tablet in the treatment of proliferative diabetic retinopathy. Evidence-Based Complementary Altern Medicine: eCAM (2021) 2021:6642600. doi: 10.1155/2021/6642600 PMC7954618 33747106

[72] Hua YF . Effect of retinal photocoagulation combined with hexue mingmu tablet on diabetic retinopathy of stage II and IV. J Chin Pract Diagnosis Ther (2012) 26(01):29–30. doi: CNKI:41-1400/R.20120110.1036.012

[73] Chen XL Tao LM . Clinical observation of calcium dobesilate and hexuemingmu tablets in treatment of nonproliferative diabetic retinopathy. Int Eye Sci (2013) 13(01):101–3. doi: 10.3980/j.issn.1672-5123.2013.01.27

[74] Xi YJ . Exploration ofthe major pharmacological effect and clinical positioning of hexuemingmu tablets against retinal degenerative diseases based on the integration strategy. [master's thesis]. Tianjin (IL): Tianjin University of Chinese Medicine (2022).

[75] Long P Yan W Liu J Li M Chen T Zhang Z . Therapeutic effect of traditional Chinese medicine on a rat model of branch retinal vein occlusion. J Ophthalmol (2019) 2019:9521379. doi: 10.1155/2019/9521379 30906588 PMC6398022

[76] Liu WN Su Y Xia YT Yan XL Li L Xiao YP . Efficacy and safety evaluation of shuangdan mingmu capsule in the treatment of type 2 diabetic retinopathy. China J Chin Ophthalmol (2022) 32(05):348–53. doi: 10.13444/j.cnki.zgzyykzz.2022.05.003

[77] Peng J Pan K Liu ZR Qin YH Peng QH . Effects of shuangdan mingmu capsule on the expressions of VEGF in the retina of diabetic rat models. Int Eye Sci (2018) 18(11):1958–62. doi: 10.3980/j.issn.1672-5123.2018.11.03

[78] Zhao HQ Fu CJ Meng P Wang YH Qin YH Zhang XL . Study on screening of effective components in shuangdan mingmu capsules inInhibiting proliferation of human umbilical vein endothelial cells. Chin J Inf Traditional Chin Med (2019) 26(07):48–51. doi: 10.3969/j.issn.1005-5304.2019.07.012

[79] Zhao YW Fu CJ Yan JC Nie FJ Chen JY Zheng XB . Effects of shuangdan mingmu capsules on blood rheology and retinal microvasculature in rats with diabetic retinopathy. Recent Adv Ophthalmol (2022) 42(05):342–6. doi: 10.13389/j.cnki.rao.2022.0069

[80] Wang J Han X Li L Han S Zhao Y Cui ZL . Treating different disease with same method in traditional Chinese medicine:mechanisms of eclipta albaglossy privet fruit medicinal pair in treating diabetic nephropathy and diabetic retinopathy based on network pharmacology. J Tianjin Univ Traditional Chin Med (2021) 40(03):374–83. doi: 10.11656/j.issn.1673-9043.2021.03.21

[81] Lin S Shi Q Ge Z Liu Y Cao Y Yang Y . Efficacy and safety of traditional Chinese medicine injections for heart failure with reduced ejection fraction: A Bayesian network meta-analysis of randomized controlled trials. Front Pharmacol (2021) 12:659707. doi: 10.3389/fphar.2021.659707 PMC8669995 34916929

[82] Moher D Hopewell S Schulz KF Montori V Gøtzsche PC Devereaux PJ . CONSORT 2010 explanation and elaboration: updated guidelines for reporting parallel group randomised trials. Int J Surg (2010) 10(1):28–55. doi: 10.1016/j.ijsu.2011.10.001 22036893

[83] Chan AW Tetzlaff JM Altman DG Laupacis A Gøtzsche PC Krleža-Jerić K . SPIRIT 2013 statement: defining standard protocol items for clinical trials. Ann Internal Med (2013) 158(3):200–7. doi: 10.7326/0003-4819-158-3-201302050-00583 PMC5114123 23295957

[84] Cheng CW Wu TX Shang HC Li YP Altman DG Moher D . CONSORT extension for Chinese herbal medicine formulas 2017: Recommendations, explanation, and elaboration. Ann Internal Med (2017) 167(2):112–21. doi: 10.7326/M16-2977 28654980

