# Supplementary material for: The comparative effects of oral Chinese patent medicines in non-proliferative diabetic retinopathy: A Bayesian network meta-analysis of randomized controlled trials
Source: Front Endocrinol (Lausanne). 2023 Apr 3;14:1144290. doi: 10.3389/fendo.2023.1144290 (PMC10106679; doi:10.3389/fendo.2023.1144290)
Supplement: Supplementary file 1 [file Table_1.docx]

Supplementary Material

The comparative effects of oral Chinese patent medicine in non-proliferative diabetic retinopathy: A Bayesian network meta-analysis of randomized controlled trials

Ziqiang Liu^1†^, Yunru Chen^2†^, Chuanhong Jie^1*^, Jianwei Wang^1^, Yu Deng^1^, Xiaoyu Hou^1^, Yuanyuan Li^1^, Wenjing Cai^1^

*** Correspondence:** Chuanhong Jie: [jiechuanhong@163.com](mailto:jiechuanhong@163.com)

# Supplementary File S1: PRISMA checklist for network meta-analysis.

|  | **Section/topic** | **#** | **Checklist item** | **Reported on page #** |
| --- | --- | --- | --- | --- |
|  | **TITLE** | | |  |
|  | Title | 1 | Identify the report as a systematic review incorporating a network meta-analysis (or related form of meta-analysis). | 1 |
|  | **ABSTRACT** | | |  |
|  | Structured summary | 2 | Provide a structured summary including, as applicable:  Background: main objectives  Methods: data sources; study eligibility criteria, participants, and interventions; study appraisal; and synthesis methods, such as network meta-analysis.  Results: number of studies and participants identified; summary estimates with corresponding confidence/credible intervals; treatment rankings may also be discussed. Authors may choose to summarize pairwise comparisons against a chosen treatment included in their analyses for brevity.  Discussion/Conclusions: limitations; conclusions and implications of findings.  Other: primary source of funding; systematic review registration number with registry name. | 1-2 |
|  | **INTRODUCTION** | | |  |
|  | Rationale | 3 | Describe the rationale for the review in the context of what is already known, including mention of why a network meta-analysis has been conducted. | 3 |
|  | Objectives | 4 | Provide an explicit statement of questions being addressed with reference to participants, interventions, comparisons, outcomes, and study design (PICOS). | 3-4 |
|  | **METHODS** | | |  |
|  | Protocol and registration | 5 | Indicate if a review protocol exists and if and where it can be accessed (e.g., Web address), and, if available, provide registration information including registration number. | 2 |
|  | Eligibility criteria | 6 | Specify study characteristics (e.g., PICOS, length of follow-up) and report characteristics (e.g., years considered, language, publication status) used as criteria for eligibility, giving rationale. Clearly describe eligible treatments included in the treatment network and note whether any have been clustered or merged into the same node (with justification). | 4 |
|  | Information sources | 7 | Describe all information sources (e.g., databases with dates of coverage, contact with study authors to identify additional studies) in the search and date last searched. | 4 |
|  | Search | 8 | Present full electronic search strategy for at least one database, including any limits used, such that it could be repeated. | S2 |
|  | Study selection | 9 | State the process for selecting studies (i.e., screening, eligibility, included in systematic review, and, if applicable, included in the meta-analysis). | 4 |
|  | Data collection process | 10 | Describe method of data extraction from reports (e.g., piloted forms, independently, in duplicate) and any processes for obtaining and confirming data from investigators. | 5 |
|  | Data items | 11 | List and define all variables for which data were sought (e.g., PICOS, funding sources) and any assumptions and simplifications made. | 4 |
|  | Geometry of the network | S1 | Describe methods used to explore the geometry of the treatment network under study and potential biases related to it. This should include how the evidence base has been graphically summarized for presentation, and what characteristics were compiled and used to describe the evidence base to readers. | 5 |
|  | Risk of bias within individual studies | 12 | Describe methods used for assessing risk of bias of individual studies (including specification of whether this was done at the study or outcome level), and how this information is to be used in any data synthesis. | 5 |
|  | Summary measures | 13 | State the principal summary measures (e.g., risk ratio, difference in means). Also describe the use of additional summary measures assessed, such as treatment rankings and surface under the cumulative ranking curve (SUCRA) values, as well as modified approaches used to present summary findings from meta-analyses. | 5 |
|  | Planned methods of analysis | 14 | Describe the methods of handling data and combining results of studies for each network meta-analysis. This should include, but not be limited to: Handling of multigroup trials; Selection of variance structure; Selection of prior distributions in Bayesian analyses; and Assessment of model fit. | 5 |
|  | Assessment of inconsistency | S2 | Describe the statistical methods used to evaluate the agreement of direct and indirect evidence in the treatment network(s) studied. Describe efforts taken to address its presence when found. | 5 |
|  | Risk of bias across studies | 15 | Specify any assessment of risk of bias that may affect the cumulative evidence (e.g., publication bias, selective reporting within studies) | 5 |
|  | Additional analyses | 16 | Describe methods of additional analyses if done, indicating which were prespecified. This may include, but not be limited to, the following: Sensitivity or subgroup analyses; Meta-regression analyses; Alternative formulations of the treatment network; and Use of alternative prior distributions for Bayesian analyses (if applicable). | 5 |
|  | **RESULTS** | | |  |
|  | Study selection | 17 | Give numbers of studies screened, assessed for eligibility, and included in the review, with reasons for exclusions at each stage, ideally with a flow diagram. | 5-6 |
|  | Presentation of network structure | S3 | Provide a network graph of the included studies to enable visualization of the geometry of the treatment network. | 6 |
|  | Summary of network geometry | S4 | Provide a brief overview of characteristics of the treatment network. This may include commentary on the abundance of trials and randomized patients for the different interventions and pairwise comparisons in the network, gaps of evidence in the treatment network, and potential biases reflected by the network structure. | 6 |
|  | Study characteristics | 18 | For each study, present characteristics for which data were extracted (e.g., study size, PICOS, follow-up period) and provide the citations. | 6 |
|  | Risk of bias within studies | 19 | Present data on risk of bias of each study and, if available, any outcome level assessment. | 6-7 |
|  | Results of individual studies | 20 | For all outcomes considered (benefits or harms), present, for each study: 1) simple summary data for each intervention group, and 2) effect estimates and confidence intervals. Modified approaches may be needed to deal with information from larger networks. | 7-9 |
|  | Synthesis of results | 21 | Present results of each meta-analysis done, including confidence/credible intervals. In larger networks, authors may focus on comparisons versus a particular comparator (e.g., placebo or standard care), with full findings presented in an appendix. League tables and forest plots may be considered to summarize pairwise comparisons. If additional summary measures were explored (such as treatment rankings), these should also be presented. | 7-9 |
|  | Exploration for inconsistency | S5 | Describe results from investigations of inconsistency. This may include such information as measures of model fit to compare consistency and inconsistency models, P values from statistical tests, or summary of inconsistency estimates from different parts of the treatment network. |  |
|  | Risk of bias across studies | 22 | Present results of any assessment of risk of bias across studies for the evidence base being studied. | 9 |
|  | Results of additional analyses | 23 | Give results of additional analyses, if done (e.g., sensitivity or subgroup analyses, meta-regression  analyses, alternative network geometries studied, alternative choice of prior distributions for  Bayesian analyses, and so forth). | 9 |
|  | **DISCUSSION** | | |  |
|  | Summary of evidence | 24 | Summarize the main findings, including the strength of evidence for each main outcome; consider their relevance to key groups (e.g., health care providers, researchers, and policymakers). | 9-12 |
|  | Limitations | 25 | Discuss limitations at study and outcome level (e.g., risk of bias), and at review level (e.g., incomplete retrieval of identified research, reporting bias). Comment on the validity of the assumptions, such as transitivity and consistency. Comment on any concerns regarding network geometry (e.g., avoidance of certain comparisons). | 12 |
|  | Conclusions | 26 | Provide a general interpretation of the results in the context of other evidence, and implications for future research. | 12-13 |
|  | **FUNDING** | | |  |
|  | Funding | 27 | Describe sources of funding for the systematic review and other support (e.g., supply of data); role of funders for the systematic review. This should also include information regarding whether funding has been received from manufacturers of treatments in the network and/or whether some of the authors are content experts with professional conflicts of interest that could affect use of treatments in the network. | 13 |

# Supplementary File S2: Search strategy for network meta-analysis.

## Search strategy of China National Knowledge Infrastructure

| **No.** | **Search items** |
| --- | --- |
| #1 | SU='糖尿病视网膜病' OR SU='非增生性糖尿病性视网膜病变' OR SU='糖尿病视网膜病变' OR SU='糖尿病性视网膜病变' OR SU='非增殖性糖尿病视网膜病变' OR SU='糖网' OR SU='非增殖性糖网' OR SU='非增生性糖网' OR SU='糖尿病眼底病' OR SU='消渴目病' OR SU='消渴内障' |
| #2 | TKA ='中成药' OR TKA ='成药' OR TKA ='中医药' OR TKA ='中医' OR TKA ='中药' OR TKA ='中西医结合' OR TKA ='中西医' OR TKA ='复方血栓通' OR TKA ='复方丹参滴丸' OR TKA ='双丹明目胶囊' OR TKA ='芪明颗粒' OR TKA ='和血明目片' OR TKA ='明目地黄丸' OR TKA ='丹红化瘀口服液' |
| #3 | FT = '随机' OR FT = '对照' OR FT = '随机分配' OR FT = '临床观察' OR FT = '临床研究' OR FT = '临床试验' OR FT = '疗效观察' |
| #4 | #1 AND #2 AND #3 |

## Search strategy of Wanfang Database

| **No.** | **Search items** |
| --- | --- |
| #1 | 主题:(糖尿病视网膜病) or 主题:(非增生性糖尿病性视网膜病变) or 主题:(糖尿病视网膜病变) or 主题:(糖尿病性视网膜病变) or 主题:(非增殖性糖尿病视网膜病变) or 主题:(糖网) or 主题:(非增殖性糖网) or 主题:(非增生性糖网) or 主题:(糖尿病眼底病) or 主题:(消渴目病) or 主题:(消渴内障) |
| #2 | 主题:(中成药) or 主题:(成药) or 主题:(中医药) or 主题:(中医) or 主题:(中药) or 主题:(中西医结合) or 主题:(中西医) or 主题:(复方血栓通) or 主题:(复方丹参滴丸) or 主题:(双丹明目胶囊) or 主题:(芪明颗粒) or 主题:(和血明目片) or 主题:(明目地黄丸) or 主题:(丹红化瘀口服液) |
| #3 | 全部:(随机) or 全部:(对照) or 全部:(随机分配) or 全部:(临床观察) or 全部:(临床研究) or 全部:(临床试验) or 全部:(疗效观察) |
| #4 | #1 AND #2 AND #3 |

## Search strategy of Chinese Biomedical Literature Database

| **No.** | **Search items** |
| --- | --- |
| #1 | "糖尿病视网膜病"[常用字段:智能] OR "非增生性糖尿病性视网膜病变"[常用字段:智能] OR "糖尿病视网膜病变"[常用字段:智能] OR "糖尿病性视网膜病变"[常用字段:智能] OR "非增殖性糖尿病视网膜病变"[常用字段:智能] OR "糖网"[常用字段:智能] OR "非增殖性糖网"[常用字段:智能] OR "非增生性糖网"[常用字段:智能] OR "糖尿病眼底病"[常用字段:智能] OR "消渴目病"[常用字段:智能] OR "消渴内障"[常用字段:智能] |
| #2 | "中成药"[常用字段:智能] OR "成药"[常用字段:智能] OR "中医"[常用字段:智能] OR "中西医结合"[常用字段:智能] OR "中西医"[常用字段:智能] OR "中药"[常用字段:智能] OR "中医药"[常用字段:智能] OR "复方血栓通"[常用字段:智能] OR "复方丹参滴丸"[常用字段:智能] OR "芪明颗粒"[常用字段:智能] OR "和血明目片"[常用字段:智能] OR "双丹明目胶囊"[常用字段:智能] OR "明目地黄丸"[常用字段:智能] OR "丹红化瘀口服液"[常用字段:智能] |
| #3 | "随机"[常用字段:智能] OR "对照"[常用字段:智能] OR "随机分配"[常用字段:智能] OR "临床观察"[常用字段:智能] OR "临床研究"[常用字段:智能] OR "临床试验"[常用字段:智能] OR "疗效观察"[常用字段:智能] |
| #4 | #1 AND #2 AND #3 |

## Search strategy of Weipu Journal Database.

| **No.** | **Search items** |
| --- | --- |
| #1 | R="糖尿病视网膜病" OR R="非增生性糖尿病性视网膜病变" OR R="糖尿病视网膜病变" OR R="糖尿病性视网膜病变" OR R="非增殖性糖尿病视网膜病变" OR R="糖网" OR R="非增殖性糖网" OR R="非增生性糖网" OR R="糖尿病眼底病" OR R="消渴目病" OR R="消渴内障" |
| #2 | (R="中成药" OR R="成药" OR R="中医" OR R="中医药" OR R="中西医结合" OR R="中西医" OR R="中药" OR R="复方血栓通" OR R="复方丹参滴丸" OR R="双丹明目胶囊" OR R="芪明颗粒" OR R="丹红化瘀口服液" OR R="明目地黄丸" OR R="和血明目片" |
| #3 | U="随机" OR U="对照" OR U="随机分配" OR U="临床观察" OR U="临床研究" OR U="临床试验" OR U="疗效观察" |
| #4 | #1 AND #2 AND #3 |

## Search strategy of Pubmed

| **No.** | **Search items** |
| --- | --- |
| #1 | "Diabetic Retinopathy"[Mesh] |
| #2 | ((Diabetic Retinopathies[Title/Abstract]) OR (Retinopathies, Diabetic[Title/Abstract])) OR (Retinopathy, Diabetic[Title/Abstract]) |
| #3 | #1 OR #2 |
| #4 | (((((((Compound Xueshuantong capsule[Title/Abstract])) OR (Compound Danshen Dripping Pill[Title/Abstract])) OR (Shuangdan Mingmu Capsule[Title/Abstract])) OR (Hexuemingmu tablet[Title/Abstract])) OR (Qiming granule[Title/Abstract])) OR (Mingmu Dihuang pill[Title/Abstract])) OR (Danhong Huayu Koufuye[Title/Abstract]) |
| #5 | (((("Medicine, Traditional"[Mesh]) OR ((((((((((((((((Traditional Medicine[Title/Abstract]) OR (Home Remedies[Title/Abstract])) OR (Home Remedy[Title/Abstract])) OR (Remedies, Home[Title/Abstract])) OR (Remedy, Home[Title/Abstract])) OR (Medicine, Primitive[Title/Abstract])) OR (Primitive Medicine[Title/Abstract])) OR (Medicine, Folk[Title/Abstract])) OR (Folk Medicine[Title/Abstract])) OR (Medicine, Indigenous[Title/Abstract])) OR (Indigenous Medicine[Title/Abstract])) OR (Folk Remedies[Title/Abstract])) OR (Folk Remedy[Title/Abstract])) OR (Remedies, Folk[Title/Abstract])) OR (Remedy, Folk[Title/Abstract])) OR (Ethnomedicine[Title/Abstract]))) |
| #6 | (("Medicine, Chinese Traditional"[Mesh]) OR (((((((((((((Traditional Chinese Medicine[Title/Abstract]) OR (Chung I Hsueh[Title/Abstract])) OR (Hsueh, Chung I[Title/Abstract])) OR (Zhong Yi Xue[Title/Abstract])) OR (Chinese Traditional Medicine[Title/Abstract])) OR (Chinese Medicine, Traditional[Title/Abstract])) OR (Traditional Tongue Diagnosis[Title/Abstract])) OR (Tongue Diagnoses, Traditional[Title/Abstract])) OR (Tongue Diagnosis, Traditional[Title/Abstract])) OR (Traditional Tongue Diagnoses[Title/Abstract])) OR (Traditional Tongue Assessment[Title/Abstract])) OR (Tongue Assessment, Traditional[Title/Abstract])) OR (Traditional Tongue Assessments[Title/Abstract])))) |
| #7 | ("Plants"[Mesh]) OR (plant[Title/Abstract]) |
| #8 | ((((((((Chinese patent medicine[Title/Abstract]) OR (patent medicine[Title/Abstract])) OR (Chinese medicine[Title/Abstract])) OR (integrated Chinese[Title/Abstract] AND western medicine[Title/Abstract])) OR (Herb[Title/Abstract])) OR (Medicinal[Title/Abstract])) OR (Complementary[Title/Abstract])) OR (alternative[Title/Abstract])) |
| #9 | #4 OR #5 OR #6 OR #7 OR #8 |
| #10 | ((((((((randomized[Title/Abstract]) OR (placebo[Title/Abstract])) OR (randomly[Title/Abstract])) OR (trial[Title/Abstract])) OR (groups[Title/Abstract])) OR ("Pragmatic Clinical Trial" [Publication Type])) OR ("Equivalence Trial" [Publication Type])) OR ("Controlled Clinical Trial" [Publication Type])) OR ("Randomized Controlled Trial" [Publication Type]) |
| #11 | #3 AND #9 AND #10 |

## Search strategy of Embase

| **No.** | **Search items** |
| --- | --- |
| #1 | 'diabetic retinopathy'/exp |
| #2 | 'diabetic retinopathies':ab,ti OR 'retinopathies, diabetic':ab,ti OR 'retinopathy, diabetic':ab,ti |
| #3 | #1 OR #2 |
| #4 | 'chinese medicine'/exp |
| #5 | 'traditional medicine'/exp |
| #6 | 'medicine, traditional':ab,ti OR 'home remedies':ab,ti OR 'home remedy':ab,ti OR 'remedies, home':ab,ti OR 'remedy, home':ab,ti OR 'medicine, primitive':ab,ti OR 'primitive medicine':ab,ti OR 'medicine, folk':ab,ti OR 'folk medicine':ab,ti OR 'medicine, indigenous':ab,ti OR 'indigenous medicine':ab,ti OR 'folk remedies':ab,ti OR 'folk remedy':ab,ti OR 'remedies, folk':ab,ti OR 'remedy, folk':ab,ti OR 'ethnomedicine':ab,ti OR 'medicine, chinese traditional':ab,ti OR 'traditional chinese medicine':ab,ti OR 'chung i hsueh':ab,ti OR 'hsueh, chung i':ab,ti OR 'traditional medicine, chinese':ab,ti OR 'zhong yi xue':ab,ti OR 'chinese traditional medicine':ab,ti OR 'chinese medicine, traditional':ab,ti OR 'traditional tongue diagnosis':ab,ti OR 'tongue diagnoses, traditional':ab,ti OR 'tongue diagnosis, traditional':ab,ti OR 'traditional tongue diagnoses':ab,ti OR 'traditional tongue assessment':ab,ti OR 'tongue assessment, traditional':ab,ti OR 'traditional tongue assessments':ab,ti |
| #7 | #4 OR #5 OR #6 |
| #8 | 'plant'/exp |
| #9 | 'plants':ab,ti |
| #10 | #8 OR #9 |
| #11 | 'herb'/exp |
| #12 | 'chinese patent medicine':ab,ti OR 'patent medicine':ab,ti OR 'medicinal':ab,ti OR 'integrated chinese and western medicine':ab,ti OR 'complementary':ab,ti OR 'alternative':ab,ti |
| #13 | 'compound xueshuantong capsule':ab,ti OR 'compound danshen dripping pill':ab,ti OR 'shuangdan mingmu capsule':ab,ti OR 'hexuemingmu tablet':ab,ti OR 'qiming granule':ab,ti OR 'mingmu dihuang pill':ab,ti OR 'danhong huayu koufuye':ab,ti |
| #14 | #7 OR #10 OR #11 OR #12 OR #13 |
| #15 | #3 AND #14 |

## Search strategy of Cochrane Library

| **No.** | **Search items** |
| --- | --- |
| #1 | MeSH descriptor: [Diabetic Retinopathy] explode all trees |
| #2 | (Diabetic Retinopathies):ti,ab,kw or (Retinopathy, Diabetic):ti,ab,kw or (Retinopathies, Diabetic):ti,ab,kw |
| #3 | #1 OR #2 |
| #4 | MeSH descriptor: [Medicine, Traditional] explode all trees |
| #5 | (Traditional Medicine):ti,ab,kw or (Home Remedies):ti,ab,kw or (Home Remedy):ti,ab,kw or (Remedies, Home):ti,ab,kw or (Remedy, Home):ti,ab,kw or (Medicine, Primitive):ti,ab,kw or (Primitive Medicine):ti,ab,kw or (Medicine, Folk):ti,ab,kw or (Folk Medicine):ti,ab,kw or (Medicine, Indigenous):ti,ab,kw or (Indigenous Medicine):ti,ab,kw or (Folk Remedies):ti,ab,kw or (Folk Remedy):ti,ab,kw or (Remedies, Folk):ti,ab,kw or (Remedy, Folk):ti,ab,kw or (Ethnomedicine):ti,ab,kw |
| #6 | #4 OR #5 |
| #7 | MeSH descriptor: [Medicine, Chinese Traditional] explode all trees |
| #8 | (Traditional Chinese Medicine):ti,ab,kw or (Chung I Hsueh):ti,ab,kw or (Hsueh, Chung I):ti,ab,kw or (Traditional Medicine, Chinese):ti,ab,kw or (Zhong Yi Xue):ti,ab,kw or (Chinese Traditional Medicine):ti,ab,kw or (Chinese Medicine, Traditional):ti,ab,kw or (Traditional Tongue Diagnosis):ti,ab,kw or (Tongue Diagnoses, Traditional):ti,ab,kw or (Tongue Diagnosis, Traditional):ti,ab,kw or (Traditional Tongue Diagnoses):ti,ab,kw or (Traditional Tongue Assessment):ti,ab,kw or (Tongue Assessment, Traditional):ti,ab,kw or (Traditional Tongue Assessments):ti,ab,kw |
| #9 | #7 OR #8 |
| #10 | MeSH descriptor: [Plants] explode all trees |
| #11 | (plant):ti,ab,kw |
| #12 | #10 OR #11 |
| #13 | (Chinese patent medicine):ti,ab,kw or (patent medicine):ti,ab,kw or (Chinese medicine):ti,ab,kw or (integrated Chinese and western medicine):ti,ab,kw or (herb):ti,ab,kw or (Medicinal):ti,ab,kw or (Complementary):ti,ab,kw or (alternative):ti,ab,kw |
| #14 | (Compound Xueshuantong capsule):ti,ab,kw or (Compound Danshen Dripping Pill):ti,ab,kw or (Shuangdan Mingmu Capsule):ti,ab,kw or (Hexuemingmu tablet):ti,ab,kw or (Qiming granule):ti,ab,kw or (Mingmu Dihuang pill):ti,ab,kw or (Danhong Huayu Koufuye):ti,ab,kw |
| #15 | #6 OR #9 OR #12 OR #13 OR #14 |
| #16 | #3 AND #15 |

# Supplementary File S3: Details of the included OCPMs.

| **Name of OCPMs** | **Manufacturer** | **Species/Raw materials** | **Scientific name of Plant or Animal** | **Indication** | **Execution standards** | **Mechanism** |
| --- | --- | --- | --- | --- | --- | --- |
| Compound Xueshuantong Capsule | Guangdong Zhongsheng Pharmaceutical Co., Ltd. | Sanchi, milkvetch root, Dan-shen Root, figwort root. | *Panax notoginseng* (Burkill) F.H.Chen [Araliaceae], *Astragalus mongholicus* Bunge [Fabaceae], *Salvia miltiorrhiza* Bunge [Lamiaceae], *Scrophularia ningpoensis* Hemsl. [Scrophulariaceae]. | Promoting blood circulation, removing blood stasis; nourishing qi and yin. | Y-National Pharmaceutical Standard: Z20030017. | CXC can reduce retinal damage by reducing erythrocyte aggregation and lowering plasma viscosity, as well as inhibiting aldose reductase activity, controlling high expression of VEGF, intercellular cell adhesion molecule-1, and endothelin-1 and low expression of pigment epithelium-derived factor. |
| Compound Danshen Dripping Pill | Tianjin Tasly Pharmaceutical Co., Ltd. | Dan-shen Root, Sanchi, Synthetic Borneol. | *Salvia miltiorrhiza* Bunge [Lamiaceae], *Panax notoginseng* (Burkill) F.H.Chen [Araliaceae], Borneolum Syntheticum. | Promoting blood circulation for removing blood stasis; regulating qi-flowing for relieving pain. | Y-National Pharmaceutical Standard: Z10950111. | CDDP can improve retinal vascular and neurological function by inhibiting inflammation, oxidative stress, and apoptosis, reducing vascular endothelial cell damage and increasing retinal thickness. |
| Shuangdan Mingmu Capsule | Beijing Qhuang Pharmaceutical Co., Ltd. | glossy privet fruit, yerbadetajo herb, Sanchi, glabrous greenbrier rhizome, twotoothed achyranthes root, Dan-shen Root, asiatic cornelian cherry fruit, common yam rhizome, tree peony bark, Poria cocos, oriental waterplantain rhizome. | *Ligustrum lucidum* W.T.Aiton [Oleaceae], *Eclipta prostrata* (L.) L. [Asteraceae], *Panax notoginseng* (Burkill) F.H.Chen [Araliaceae], *Smilax glabra* Roxb. [Smilacaceae], *Achyranthes bidentata* Blume [Amaranthaceae], *Salvia miltiorrhiza* Bunge [Lamiaceae], *Cornus officinalis* Siebold & Zucc. [Cornaceae], *Dioscorea oppositifolia* L. [Dioscoreaceae], *Paeonia×suffruticosa* Andrews [Paeoniaceae], Poria cocos, *Alisma plantago-aquatica* subsp. orientale (Sam.) Sam. [Alismataceae]. | Nourishing kidneys and liver; promoting blood and brightening eyesight. | Y-National Pharmaceutical Standard: Z20080062. | SDMMC can improve glucose dyslipidemia and blood rheology, and reduce oxidative stress and the expression of VEGF. |
| Qiming Qranule | Zhejiang Wansheng Pharmaceutical Co., Ltd. | milkvetch root, kudzuvine root, rehmannia root, lycii fructus, cassia seed, leonuri fructus, cattail pollen, leech. | *Astragalus mongholicus* Bunge [Fabaceae], *Pueraria montana var. lobata* (Willd.) Maesen & S.M.Almeida ex Sanjappa & Predeep [Fabaceae], *Rehmannia glutinosa* (Gaertn.) DC. [Orobanchaceae], *Lycium barbarum* L. [Solanaceae], *Senna tora* (L.) Roxb. [Fabaceae], *Leonurus japonicus* Houtt. [Lamiaceae], *Typha angustifolia* L. [Typhaceae], leech. | Enhancing qi while nourishing fluid; nourishing liver and kidney; clearing collaterals and brightening eyes. | Y-National Pharmaceutical Standard: Z20090036. | QG can improve retinal microcirculation, reduce capillary permeability and increase blood perfusion, to improve retinal function. |
| Hexuemingmu Tablet | Xi 'an Beilin Pharmaceutical Co., Ltd. | cattail pollen, Dan-shen Root, rehmannia root, yerbadetajo herb, chrysanthemun flower, baical skullcap root, cassia seed, plantain seed, leonuri fructus, glossy privet fruit, common selfheal fruit-spike, gentianae radix et rhizoma, turmeric root tuber, common scouring rush herb, red peony root, tree peony bark, chinese angelica root, chuanxiong rhizoma. | *Typha angustifolia* L. [Typhaceae], *Salvia miltiorrhiza* Bunge [Lamiaceae], *Rehmannia glutinosa* (Gaertn.) DC. [Orobanchaceae], *Eclipta prostrata* (L.) L. [Asteraceae], *Chrysanthemum × morifolium* (Ramat.) Hemsl. [Asteraceae], *Scutellaria baicalensis* Georgi [Lamiaceae], *Senna tora* (L.) Roxb. [Fabaceae], *Plantago asiatica* L. [Plantaginaceae], *Leonurus japonicus* Houtt. [Lamiaceae], *Ligustrum lucidum* W.T.Aiton [Oleaceae], *Prunella vulgaris* L. [Lamiaceae], *Gentiana scabra* Bunge [Gentianaceae], *Curcuma aromatica* Salisb. [Zingiberaceae], *Equisetum hyemale* L. [Equisetaceae], *Paeonia anomala subsp. veitchii* (Lynch) D.Y.Hong & K.Y.Pan [Paeoniaceae], *Paeonia×suffruticosa* Andrews [Paeoniaceae], *Angelica sinensis* (Oliv.) Diels [Apiaceae], *Conioselinum anthriscoides* 'Chuanxiong' [Apiaceae]. | Cooling blood to stop bleeding; nourishing Yin and removing blood stasis; nourishing liver and improving eyesight. | Y-National Pharmaceutical Standard: Z20073062. | HXMMT can effectively reduce retinal damage through antioxidant, anti-inflammatory, and anti-angiogenic properties. |
| Danhong Huayu Koufuye | Guangzhou Baiyun Mountain Hutchison Whampoa Traditional Chinese Medicine Co., Ltd. | Dan-shen Root, chinese angelica root, chuanxiong rhizoma, persicae semen, carthami flos, bupleuri radix, aurantii fructus. | *Salvia miltiorrhiza* Bunge [Lamiaceae], *Angelica sinensis* (Oliv.) Diels [Apiaceae], *Conioselinum anthriscoides* 'Chuanxiong' [Apiaceae], *Prunus persica* (L.) Batsch [Rosaceae], *Carthamus tinctorius* L. [Asteraceae], *Bupleurum chinense* DC. [Apiaceae], *Citrus × aurantium* L. [Rutaceae]. | Promoting blood circulation and removing blood stasis; promoting qi circulation and removing obstruction in the collateral(s). | Y-National Pharmaceutical Standard: Z10960051. | DHHYK can protect endothelial cells and inhibit neovascularization through antioxidant and anti-inflammatory activities. |
| Mingmu Dihuang Pill | Beijing Tong Ren Tang Co., Ltd. | rehmannia root, corni fructus,tree peony bark, common yam rhizome, Poria cocos, oriental waterplantain rhizome, lycii fructus, chrysanthemun flower, chinese angelica root, paeoniae radix alba, tribuli fructus, Concha Haliotidis. | *Rehmannia glutinosa* (Gaertn.) DC. [Orobanchaceae], *Cornus officinalis* Siebold & Zucc. [Cornaceae], *Paeonia×suffruticosa* Andrews [Paeoniaceae], *Dioscorea oppositifolia* L. [Dioscoreaceae], Poria cocos, *Alisma plantago-aquatica* subsp. orientale (Sam.) Sam. [Alismataceae], *Lycium barbarum* L. [Solanaceae], *Chrysanthemum × morifolium* (Ramat.) Hemsl. [Asteraceae], *Angelica sinensis* (Oliv.) Diels [Apiaceae], *Paeonia lactiflor*a Pall. [Paeoniaceae], *Tribulus terrestris* L. [Zygophyllaceae], Concha Haliotidis. | Nourishing the kidney; nourishing the liver; brightening the eyes. | Y-National Pharmaceutical Standard: Z11020166. | MMDHP can improve inflammation and oxidative stress state, inhibit angiogenesis, and improve retinal function. |

# Supplementary File S4: The forest plots of Pairwise Meta-Analysis of seven outcomes.

A B
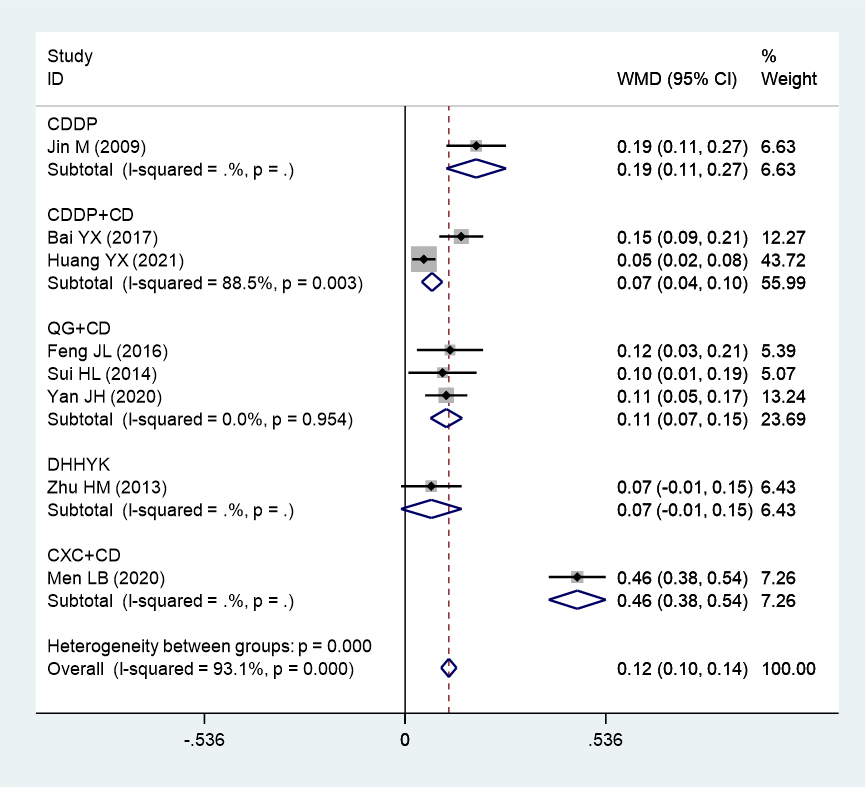


C
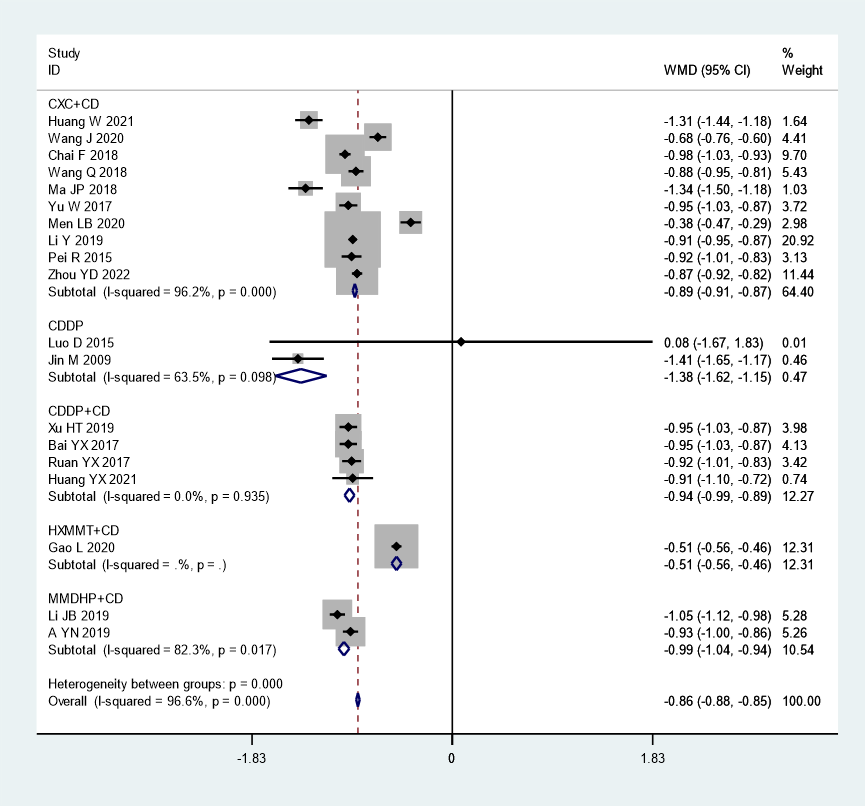
 D

E F
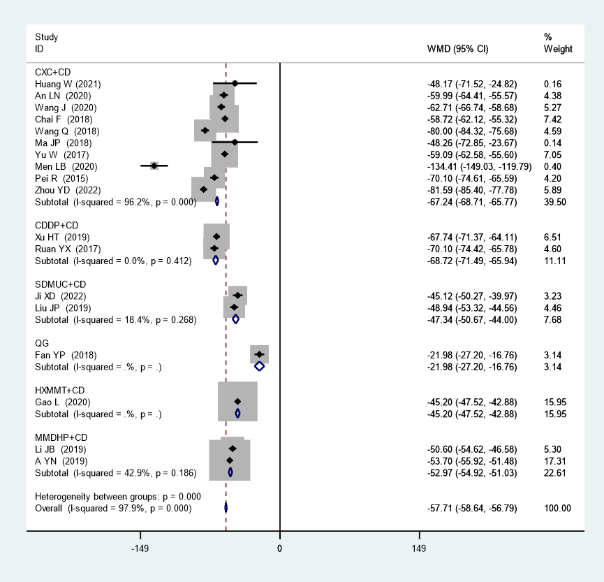


G
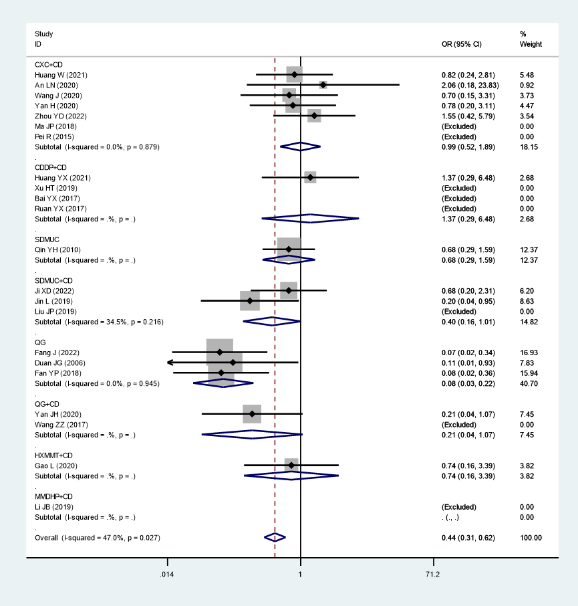


**Figure S4** The forest plot Pairwise Meta-Analysis of seven outcomes. **(A)** Clinical effective rate; **(B)** Best corrected visual acuity; **(C)** Visual field gray value; **(D)** Microaneurysm volume; **(E)** Hemorrhage area; **(F)** Macular thickness; **(G)** Adverse drug reactions. CD, calcium dobesilate; CXC, Compound Xueshuantong Capsule; CDDP, Compound Danshen Dripping Pill; SDMUC, Shuangdan Mingmu Capsule; QG, Qiming Granule; HXMMT, Hexuemingmu Tablet; DHHYK, Danhong Huayu Koufuye; MMDHP, Mingmu Dihuang Pill.

# Supplementary File S5: Detailed information of heterogeneity analysis

## Table S5-1 Clinical effective rate

| **Clinical effective rate** | **N（studies）** | **OR 95%CI** | ***P*** | **I-squared** | ***P*** |
| --- | --- | --- | --- | --- | --- |
| CXC | 1 | 2.87 (0.92, 8.90) | 0.069 | - | - |
| CXC+CD | 13 | 3.92 (2.87, 5.34) | 0.000 | 0.0% | 0.996 |
| CDDP | 1 | 5.67 (1.80, 17.93) | 0.003 | - | - |
| CDDP+CD | 5 | 7.49 (3.40, 16.50) | 0.000 | 0.0% | 0.666 |
| DHHYK | 1 | 2.40 (1.00, 5.76) | 0.050 | - | - |
| HXMMT+CD | 2 | 3.22 (1.96, 5.29) | 0.000 | 0.0% | 0.954 |
| MMDHP+CD | 1 | 2.96 (0.74, 11.82) | 0.125 | - | - |
| QG | 4 | 3.57 (2.32, 5.49) | 0.000 | **83.0%** | **0.001** |
| QG+CD | 4 | 5.01 (2.55, 9.87) | 0.000 | 0.0% | 0.844 |
| SDMUC | 1 | 3.71 (2.40, 5.73) | 0.000 | - | - |
| SDMUC+CD | 3 | 4.45 (1.88, 10.63) | 0.001 | 0.0% | 0.952 |

## Table S5-2 Best corrected visual acuity

| **Visual acuity** | **N（studies）** | **MD 95%CI** | ***P*** | **I-squared** | ***P*** |
| --- | --- | --- | --- | --- | --- |
| CXC+CD | 1 | 0.46 (0.38, 0.54) | 0.000 | - | - |
| CDDP | 1 | 0.19 (0.11, 0.27) | 0.000 | - | - |
| CDDP+CD | 2 | 0.07 (0.04, 0.10) | 0.000 | 88.5% | 0.003 |
| DHHYK | 1 | 0.07 (-0.01, 0.15) | 0.090 | - | - |
| QG+CD | 3 | 0.11 (0.07, 0.15) | 0.000 | 0.0% | 0.954 |

## Table S5-3 Visual field gray value

| **Visual field gray value** | **N（studies）** | **MD 95%CI** | ***P*** | **I-squared** | ***P*** |
| --- | --- | --- | --- | --- | --- |
| CXC+CD | 10 | -0.89 (-0.91, -0.87) | 0.000 | **96.2%** | **0.000** |
| CDDP | 2 | -1.38 (-1.62, -1.15) | 0.000 | 63.5% | 0.098 |
| CDDP+CD | 4 | -0.94 (-0.99, -0.89) | 0.000 | 0.0% | 0.935 |
| HXMMT+CD | 1 | -0.51 (-0.56, -0.46) | 0.000 | - | - |
| MMDHP+CD | 2 | -0.99 (-1.04, -0.94) | 0.000 | 82.3% | 0.017 |

## Table S5-4 Microaneurysm volume

| **Microaneurysm volume** | **N（studies）** | **MD 95%CI** | ***P*** | **I-squared** | ***P*** |
| --- | --- | --- | --- | --- | --- |
| CXC+CD | 7 | -3.51 (-3.95, -3.08) | 0.000 | 0.0% | 0.891 |
| CDDP+CD | 4 | -3.06 (-3.41, -2.71) | 0.000 | 0.0% | 0.967 |
| HXMMT+CD | 1 | -4.04 (-4.81, -3.27) | 0.000 | - | - |
| MMDHP+CD | 2 | -3.18 (-3.51, -2.85) | 0.000 | 0.0% | 0.752 |

## Table S5-5 Hemorrhage area

| **Hemorrhage area** | **N（studies）** | **MD 95%CI** | ***P*** | **I-squared** | ***P*** |
| --- | --- | --- | --- | --- | --- |
| CXC+CD | 9 | -0.80 (-0.87, -0.73) | 0.000 | 35.4% | 0.135 |
| CDDP | 1 | -0.80 (-1.06, -0.54) | 0.000 | - | - |
| CDDP+CD | 3 | -0.80 (-0.89, -0.71) | 0.000 | 0.0% | 0.502 |
| HXMMT+CD | 1 | -0.56 (-0.67, -0.45) | 0.000 | - | - |
| MMDHP+CD | 2 | -0.85 (-0.92, -0.78) | 0.000 | 0.0% | 0.594 |
| SDMUC+CD | 2 | -0.91 (-1.01, -0.81) | 0.000 | 0.0% | 0.770 |

## Table S5-6 Macular thickness

| **Macular thickness** | **N（studies）** | **MD 95%CI** | ***P*** | **I-squared** | ***P*** |
| --- | --- | --- | --- | --- | --- |
| CXC+CD | 10 | -67.24 (-68.71, -65.77) | 0.000 | **96.2%** | **0.000** |
| CDDP+CD | 2 | -67.24 (-68.71, -65.77) | 0.000 | 0.0% | 0.412 |
| HXMMT+CD | 1 | -45.30 (-47.52, -42.88) | 0.000 | - | - |
| MMDHP+CD | 2 | -52.97 (-54.92, -51.03) | 0.000 | 42.9% | 0.186 |
| QG | 1 | -21.98 (-27.20, -16.76) | 0.000 | - | - |
| SDMUC+CD | 2 | -47.336 (-50.68, -44.00) | 0.000 | 18.4% | 0.268 |

## Table S5-7 ADRs

| **Macular thickness** | **N（studies）** | **MD 95%CI** | ***P*** | **I-squared** | ***P*** |
| --- | --- | --- | --- | --- | --- |
| CXC+CD | 7 | 0.99 (0.52, 1.89) | 0.979 | 0.0% | 0.879 |
| CDDP+CD | 4 | 1.37 (0.29, 6.48) | 0.695 | - | - |
| HXMMT+CD | 1 | 0.74 (0.16, 3.39) | 0.702 | - | - |
| MMDHP+CD | 1 | - | - | - | - |
| QG | 3 | 0.08 (0.03, 0.22) | 0.000 | 0.0% | 0.945 |
| QG+CD | 2 | 0.21 (0.04, 1.04) | 0.055 | - | - |
| SDMUC | 1 | 0.68 (0.30, 1.59) | 0.376 | - | - |
| SDMUC+CD | 3 | 0.40 (0.16, 1.01) | 0.053 | 34.5% | 0.216 |

# Supplementary File S6: The details of subgroup analysis

|  | **The curative time (month)** | **Study** | **I-squared** | **P** |
| --- | --- | --- | --- | --- |
| Clinical effective rate | 3m | 2 | 0.0% | 0.948 |
|  | 6m | 2 | 0.0% | 0.724 |
| Visual field gray value | >3m | 5 | 96.1% | 0.000 |
|  | ≤3m | 5 | 96.9% | 0.000 |
| Macular thickness | >3m | 5 | 89.9% | 0.000 |
|  | ≤3m | 6 | 99.8% | 0.000 |

# Supplementary File S7: The details of Sensitivity analysis for pairwise meta-analysis

## Change model

|  | **Model** | **OR/MD 95%CI** | ***P*** | **I-squared** | ***P*** |
| --- | --- | --- | --- | --- | --- |
| Clinical effective rate | Fixed | 3.57 (2.32, 5.49) | 0.000 | 83.0% | 0.001 |
|  | Random | 5.49 (1.44, 20.78) | 0.001 | 83.0% | 0.013 |
| Visual field gray value | Fixed | -0.89 (-0.91, -0.87) | 0.000 | 96.2% | 0.000 |
|  | Random | -0.91 (-1.02, -0.81) | 0.000 | 96.2% | 0.029 |
| Macular thickness | Fixed | -67.24 (-68.71, -65.77) | 0.000 | 96.2% | 0.000 |
|  | Random | -64.63 (-86.27, -42.98) | 0.000 | 99.7% | 0.000 |

## Remove any study

A B

C

**Figure S7.2** Figure of sensitivity analysis. **(A)** Clinical effective rate; **(B)** Visual field gray value; **(C)** Macular thickness.

# Supplementary File S8: The details of Sensitivity analysis for NMA meta-analysis

## Remove low-quality literature

**TABLE S8.1 Ranking probability of interventions**

| **Intervention** | **Clinical effective rate** | | **Visual acuity** | | **Visual field gray value** | | **Microaneurysm volume** | | **Hemorrhage area** | | **Macular thickness** | |
| --- | --- | --- | --- | --- | --- | --- | --- | --- | --- | --- | --- | --- |
|  | **SUCRA (%)** | **Rank** | **SUCRA (%)** | **Rank** | **SUCRA (%)** | **Rank** | **SUCRA (%)** | **Rank** | **SUCRA (%)** | **Rank** | **SUCRA (%)** | **Rank** |
| CXC+CD | 56.68 | 6 | 98.51 | 1 | 56.74 | 4 | 72.88 | 2 | 56.76 | 4 | 85.45 | 1 |
| CDDP+CD | 86.39 | 1 | 41.8 | 4 | 59.74 | 3 | 37.28 | 4 | 56.48 | 5 | 76.81 | 2 |
| QG+CD | 66.31 | 3 | 49.27 | 3 | - | - | - | - | - | - | - | - |
| SDMUC+CD | 59.96 | 4 | - | - | - | - | - | - | 86.34 | 1 | 51.12 | 4 |
| MMDHP+CD | 43.86 | 8 | - | - | 67.47 | 2 | 45.74 | 3 | 72.83 | 2 | 57.93 | 3 |
| HXMMT+CD | 40.06 | 9 | - | - | 23.7 | 5 | 94.09 | 1 | 18.93 | 6 | 49.85 | 5 |
| CXC | 40.02 | 10 | - | - | - | - | - | - | - | - | - | - |
| CDDP | 68.14 | 2 | 70.34 | 2 | 91.83 | 1 | - | - | 58.64 | 3 | - | - |
| QG | 57.8 | 5 | - | - | - | - | - | - | - | - | 25.17 | 6 |
| SDMUC | 47.29 | 7 | - | - | - | - | - | - | - | - | - | - |
| DHHYK | 30.84 | 11 | 33.85 | 5 | - | - | - | - | - | - | - | - |
| CD | 2.64 | 12 | 6.25 | 6 | 0.51 | 6 | 0 | 5 | 0 | 7 | 3.65 | 7 |


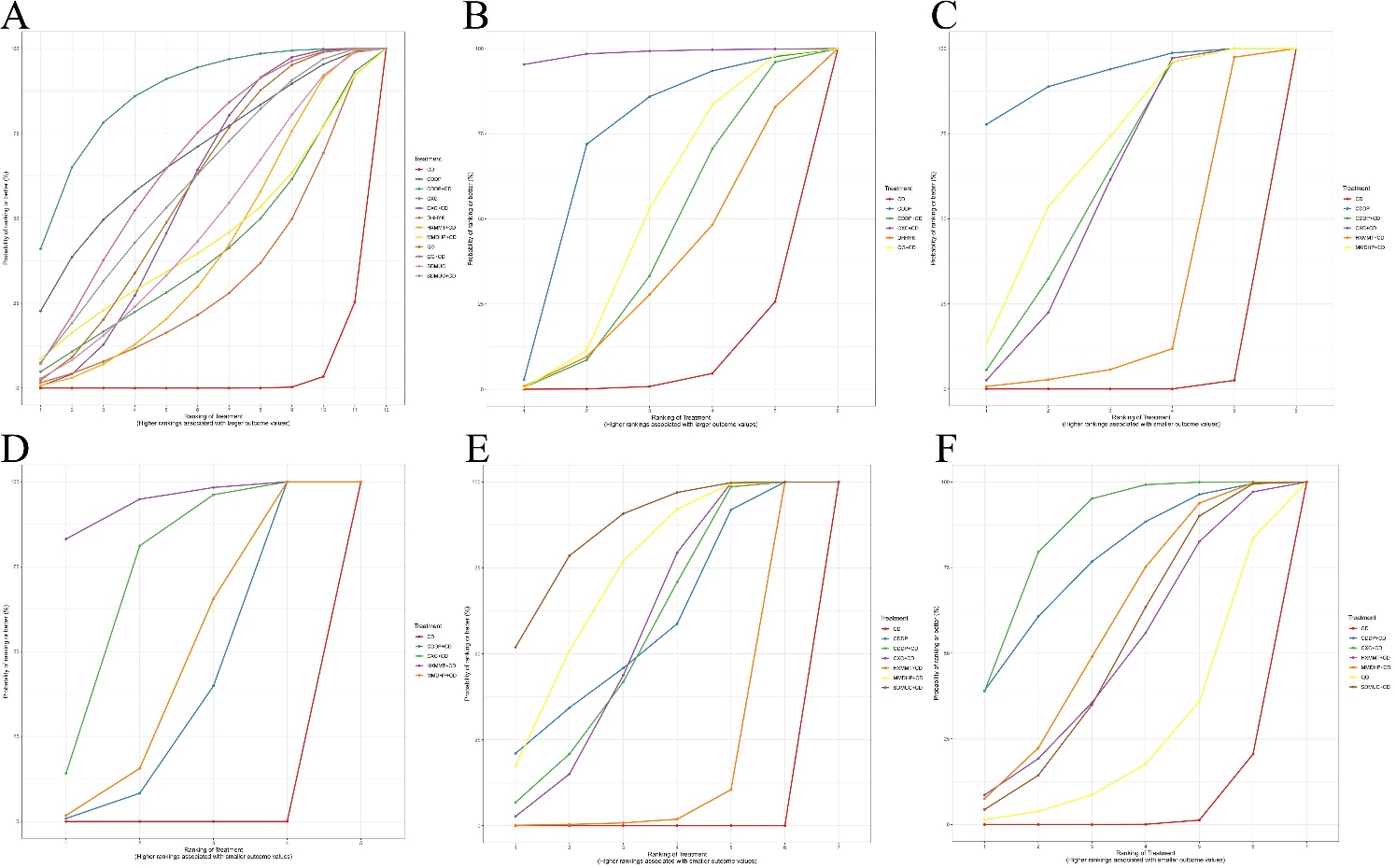


**Figure S8.1** Surface under the cumulative ranking curve (SUCRA) probabilities of different interventions for six outcomes. **(A)** Clinical effective rate; **(B)** Visual acuity; **(C)** Visual field gray value; **(D)** Microaneurysm volume; **(E)** Hemorrhage area; **(F)** Macular thickness. Note: CD, calcium dobesilate; CXC, Compound Xueshuantong Capsule; CDDP, Compound Danshen Dripping Pill; SDMUC, Shuangdan Mingmu Capsule; QG, Qiming Granule; HXMMT, Hexuemingmu Tablet; DHHYK, Danhong Huayu Koufuye; MMDHP, Mingmu Dihuang Pill.

## Remove literatures with treatment course less than 3 months

**TABLE S8.2 Ranking probability of interventions**

| **Intervention** | **Clinical effective rate** | | **Visual acuity** | | **Visual field gray value** | | **Microaneurysm volume** | | **Hemorrhage area** | | **Macular thickness** | |
| --- | --- | --- | --- | --- | --- | --- | --- | --- | --- | --- | --- | --- |
|  | **SUCRA (%)** | **Rank** | **SUCRA (%)** | **Rank** | **SUCRA (%)** | **Rank** | **SUCRA (%)** | **Rank** | **SUCRA (%)** | **Rank** | **SUCRA (%)** | **Rank** |
| CXC+CD | 52.9 | 6 | - | - | 60.67 | 3 | 71.82 | 2 | 59.86 | 3 | 86.21 | 2 |
| CDDP+CD | 81.28 | 1 | 53.26 | 3 | 53.61 | 4 | 40.3 | 4 | 58.05 | 4 | 92.08 | 1 |
| QG+CD | 60.74 | 4 | 59.97 | 2 | - | - | - | - | - | - | - | - |
| SDMUC+CD | 61.56 | 3 | - | - | - | - | - | - | 81.18 | 1 | 49.37 | 4 |
| MMDHP+CD | 43.91 | 8 | - | - | 70.23 | 2 | 44.37 | 3 | 75.11 | 2 | 56.75 | 3 |
| HXMMT+CD | 40.55 | 9 | - | - | 21.52 | 5 | 93.52 | 1 | 19.63 | 6 | 46.68 | 5 |
| CXC | - | - | - | - | - | - | - | - | - | - | - | - |
| CDDP | 70.03 | 2 | 84.85 | 1 | 93.78 | 1 | - | - | 56.16 | 5 | - | - |
| QG | 58.7 | 5 | - | - | - | - | - | - | - | - | 18.46 | 6 |
| SDMUC | 47.92 | 7 | - | - | - | - | - | - | - | - | - | - |
| DHHYK | 30.41 | 10 | 42.63 | 4 | - | - | - | - | - | - | - | - |
| CD | 2 | 11 | 9.29 | 5 | 0.18 | 6 | 0 | 5 | 0.01 | 7 | 0.46 | 7 |


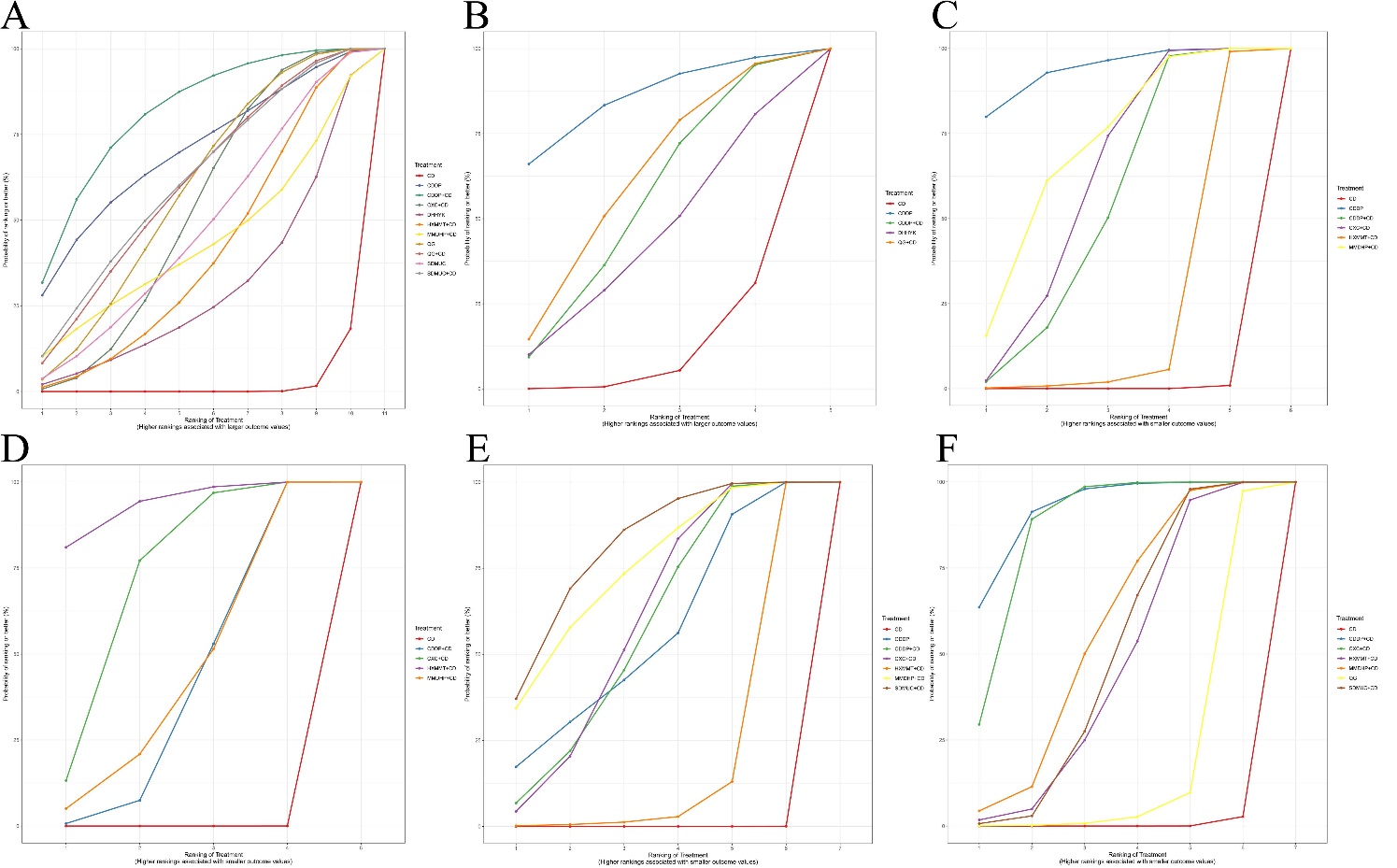


**Figure S8.2** Surface under the cumulative ranking curve (SUCRA) probabilities of different interventions for six outcomes. **(A)** Clinical effective rate; **(B)** Visual acuity; **(C)** Visual field gray value; **(D)** Microaneurysm volume; **(E)** Hemorrhage area; **(F)** Macular thickness. Note: CD, calcium dobesilate; CXC, Compound Xueshuantong Capsule; CDDP, Compound Danshen Dripping Pill; SDMUC, Shuangdan Mingmu Capsule; QG, Qiming Granule; HXMMT, Hexuemingmu Tablet; DHHYK, Danhong Huayu Koufuye; MMDHP, Mingmu Dihuang Pill.
